# Antibiotic exposure perturbs the gut microbiota and elevates mortality in honeybees

**DOI:** 10.1371/journal.pbio.2001861

**Published:** 2017-03-14

**Authors:** Kasie Raymann, Zack Shaffer, Nancy A. Moran

**Affiliations:** Department of Integrative Biology, University of Texas, Austin, Texas, United States of America; Massachusetts Institute of Technology, United States of America

## Abstract

Gut microbiomes play crucial roles in animal health, and shifts in the gut microbial community structure can have detrimental impacts on hosts. Studies with vertebrate models and human subjects suggest that antibiotic treatments greatly perturb the native gut community, thereby facilitating proliferation of pathogens. In fact, persistent infections following antibiotic treatment are a major medical issue. In apiculture, antibiotics are frequently used to prevent bacterial infections of larval bees, but the impact of antibiotic-induced dysbiosis (microbial imbalance) on bee health and susceptibility to disease has not been fully elucidated. Here, we evaluated the effects of antibiotic exposure on the size and composition of honeybee gut communities. We monitored the survivorship of bees following antibiotic treatment in order to determine if dysbiosis of the gut microbiome impacts honeybee health, and we performed experiments to determine whether antibiotic exposure increases susceptibility to infection by opportunistic pathogens. Our results show that antibiotic treatment can have persistent effects on both the size and composition of the honeybee gut microbiome. Antibiotic exposure resulted in decreased survivorship, both in the hive and in laboratory experiments in which bees were exposed to opportunistic bacterial pathogens. Together, these results suggest that dysbiosis resulting from antibiotic exposure affects bee health, in part due to increased susceptibility to ubiquitous opportunistic pathogens. Not only do our results highlight the importance of the gut microbiome in honeybee health, but they also provide insights into how antibiotic treatment affects microbial communities and host health.

## Author summary

There is growing evidence for the importance of gut microbes in animal health. Unlike most other insects, honeybees possess a highly conserved gut microbial community, which is acquired through social contact, and several results have suggested that these microbes play an important role in honeybee health. Antibiotics, which can severely disrupt gut microbial communities, are commonly used in beekeeping in several countries. However, it is unknown how antibiotic treatment affects the gut microbial communities of honeybees. Here, we evaluated the effects of antibiotic treatment on the size and composition of the honeybee gut microbiome and on honeybee health. We found that exposure to antibiotics significantly alters the honeybee gut microbial community structure and leads to decreased survivorship of honeybees in the hive, likely due to increased susceptibility to infection by opportunistic pathogens.

## Introduction

Gut microbial communities influence animal health in many ways, including synthesis of vitamins, digestion of food, defense against pathogens, and modulation of behavior, development, and immunity [1]. The gut microbial community can be disturbed by several factors: one of the most potent sources of disturbance for humans and domesticated animals is antibiotic treatment, which can severely alter community size and composition [2]. Treatment with antibiotics has also been associated with the appearance of resistant pathogens such as *Clostridium difficile* and *Salmonella enterica* [3–5]. Multiple studies have shown that reduction of gut microbial diversity occurs within a few days of ingestion of antibiotics [1,6,7], and complete recovery of initial bacterial community composition is rarely achieved [7]. In fact, it has been suggested that the overuse of antibiotics has permanently changed our microbiomes, causing an increase in “modern plagues” such as obesity, asthma, diabetes, and certain forms of cancer [8]. However, the duration and extent of antibiotic-induced disturbance in the gut microbiota remains poorly characterized, particularly at the species and strain level where the diversity of the gut community is the greatest [9,10]. Characterizing shifts in size and composition of the microbiota is particularly difficult in mammalian hosts, because of the complexity of their gut communities.

Model organisms provide opportunities to study host—microbiome interactions with a level of experimental control that is not achievable in human studies, and models can be used to understand the generality of associations between the microbiome and disease. The gut communities in social insects, such as honeybees (*Apis mellifera*), are particularly useful as models, because they share common features with mammalian gut communities. As in mammals, honeybees acquire their gut microbiota through social contact [11], in contrast to many invertebrates, which acquire gut bacteria from environmental sources. Similar to humans, the gut microbiota of honeybees is composed of host-specialized bacterial species that live only in the host gut [12] and that show considerable strain diversity within individual hosts [13]. However, in contrast to humans and other mammals, bees have a relatively simple gut microbiota, dominated by only eight core bacterial species, which comprise 95%–99% of bacteria in the gut [14,15]. Therefore, the honeybee provides a tractable system to study the function and evolution of host-associated microbial communities. Additionally, honeybees are important globally as agricultural pollinators [16]. Since 2006, the world's honeybee colonies have undergone elevated mortality, with multiple factors linked to the declines [17,18]. Several results suggest that the gut microbiome contributes to bee health [19–22]. Therefore, dysbiosis (microbial imbalance) may impact honeybee health and susceptibility to disease.

Another parallel between the microbiomes of honeybees and humans is the long history of exposure to antibiotics, a potent source of disturbance to gut communities. Antibiotic treatment of bee colonies has been widely used for over 50 y in the United States to prevent a bacterial disease of bee larvae called foulbrood (*Paenibacillus larvae*) [23–25]. The two antibiotics most commonly used by beekeepers are tetracycline (or the related compound oxytetracycline) and, since 2006, tylosin. Tetracyclines are also used for treating bacterial infections in humans and are commonly incorporated into livestock feed, resulting in the acquisition of tetracycline resistance in many bacteria, including some pathogenic taxa [26]. Likewise, the use of tetracycline in US beekeeping has resulted in an accumulation of resistance genes in microbiomes of US honeybees as compared to bumblebees or honeybees in countries that do not use antibiotics in beekeeping [27]. Widespread antibiotic resistance has been reported in *P*. *larvae*, the bacterial pathogen that causes American Foulbrood (AFB) [28], and a resistance gene (*tetL*) found in *P*. *larvae* is identical in sequence to one of the resistance loci harbored by honeybee gut symbionts [27], suggesting past horizontal transfer between commensal gut bacteria and this pathogen.

In this study, we evaluate the effects of tetracycline exposure on bee survivorship and on the size and composition of honeybee gut communities. We sampled treated bees at different time-points postexposure to determine if the microbiome recovers to pretreatment status. We monitored post-treatment survival within hives to determine if gut dysbiosis impacts honeybee health, and we tested whether antibiotic exposure increases susceptibility to infection by opportunistic pathogens present in hives. Our results show that treatment with tetracycline severely alters both the size and composition of the honeybee gut microbiome. Moreover, the perturbations caused by tetracycline treatment were still evident one week after bees were returned to their hives following exposure. Our results show that tetracycline-induced dysbiosis can decrease the survival rate of bees and suggest that this reflects increased susceptibility to opportunistic pathogens.

## Results

Adult worker bees were collected from a brood frame from a single hive. Bees were fed filter-sterilized sucrose syrup (controls) or tetracycline suspended in filter-sterilized sucrose syrup (treatments) for 5 d before being returned to the hive or maintained in the laboratory under sterile (i.e., kept only with other tetracycline-treated bees from their cohort) or exposed (i.e., with normal workers collected from their hive) recovery conditions. In order to determine how antibiotic treatment affects longevity and the size and composition of the gut microbiome, bees were censused and sampled at several time points post-treatment. The gut microbiota was assessed for total number of bacteria and for community composition using quantitative PCR and deep amplicon sequencing of a region of the bacterial 16S rRNA gene.

**Fig 1:**
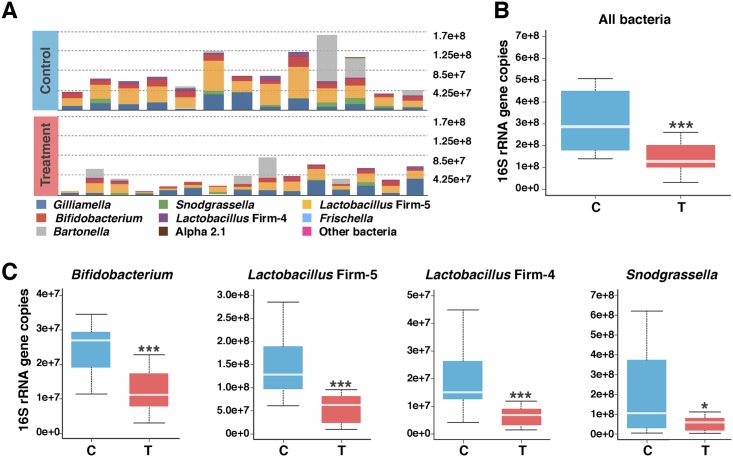
Changes in the honeybee gut microbiota after 5 d of tetracycline treatment (Day 0 post-treatment). **A)** Stacked column graph showing the abundance of bee gut bacterial species in control bees (*n* = 14) and treatment bees (*n* = 15). Abundance of the bee gut bacterial species was estimated by correcting for absolute abundance (estimated by qPCR) and taking into account rRNA operon number per genome (S1 Table). **B)** Boxplot of total bacterial 16S rRNA gene copies estimated by qPCR for control and treatment bees. **C)** Boxplots showing decreased abundance of four core gut species in treatment bees (estimated by multiplying the percent relative abundance of each species by the total bacterial 16S rRNA gene copies and correcting for rRNA operon number per genome). Box-and-whisker plots show high, low, and median values, with lower and upper edges of each box denoting first and third quartiles, respectively. * = *p* < 0.05 and *** = *p* < 0.0001, Wilcoxon rank sum tests. See S1 Data for absolute and relative abundance data.

Antibiotic treatment resulted in major changes in community size starting on the first sampling day (Day 0, before reintroduction to the hive) (Fig 1A and 1B). None of the core species was completely eradicated by tetracycline treatment, but the total bacterial abundance as well as the absolute abundance of several species decreased in treated bees (Wilcoxon test, *p* < 0.05) (Fig 1B and 1C). Of eight core bacterial species found in the honeybee gut [14], four were significantly affected by tetracycline treatment. The Gram-positive taxa, *Bifidobacterium*, *Lactobacillus* Firm-5, and *Lactobacillus* Firm-4, were the most affected (Wilcoxon test, *p* < 0.0001) (Fig 1C). The Gram-negative species, *Snodgrassella alvi*, was also reduced on Day 0 (Wilcoxon test, *p* < 0.05) (Fig 1C). Although changes in absolute abundance were seen at the first sampling time-point, no significant changes in the relative abundances of the native bacterial taxa were detected (S1 Fig).

**Fig 2:**
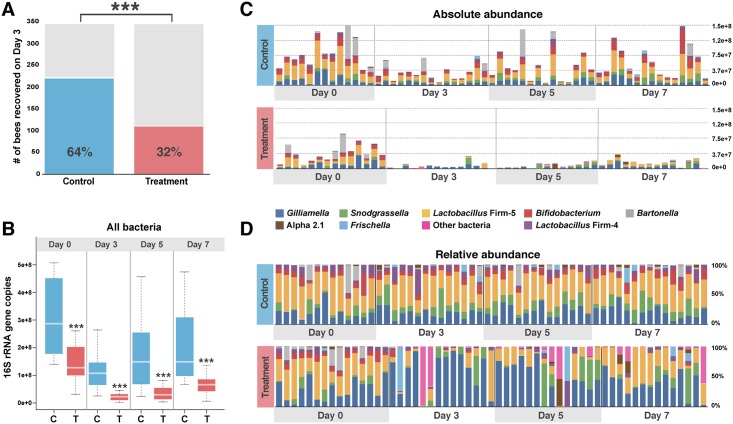
Survival rate and gut community changes for honeybees returned to the hive after tetracycline treatment. **A)** Number of workers recovered from the hive on Day 3 post-treatment (*p* < 0.0001, Chi-squared test). Similar results were obtained in a second experiment with a different hive (S2A Fig). See S2 Data for survival counts. **B-D)** Honeybee gut microbiome composition at post-treatment Days 0 (control *n* = 13, treatment *n* = 15), 3 (control *n* = 14, treatment *n* = 12), 5 (control *n* = 15, treatment *n* = 15), and 7 (control *n* = 15, treatment *n* = 14) **B)** Stacked column graph showing the absolute abundance of bacterial species present in control and treatment bees. **C)** Boxplot of total bacterial 16S rRNA gene copies for control and treatment bees post-treatment at Days 0, 3, 5, and 7. Box-and-whisker plots show high, low, and median values, with lower and upper edges of each box denoting first and third quartiles, respectively. *** = *p* < 0.0001, Wilcoxon rank sum tests. **D)** Stacked column graph showing the relative abundance of bacterial species in control and treatment bees at Days 0, 3, 5, and 7. See S1 Data for absolute and relative abundance data.

After reintroduction to the hive, bees were censused to determine effects of tetracycline treatment on longevity. Recovery of treatment bees (32%) from the hive on Day 3 post-treatment was significantly lower than the recovery of control bees (64%) (Chi-squared test, *p* < 0.0001) (Fig 2A). A replicate survival experiment performed in a different hive also indicated that tetracycline-treated bees have increased mortality in the hive (S2A Fig). We also performed laboratory recovery experiments in order to control for age, determine the effects of tetracycline on the survival of germ-free bees, and determine if the bee gut microbiome could recover with exposure to workers from their hive or without such exposure. The complementary experiments performed on bees kept in the laboratory showed that the decrease in survival rate is not due to side effects of tetracycline (S2B–S2D Fig). For bees possessing their natural microbiota, antibiotic treatment caused a decrease in survival during recovery in laboratory-kept bees, for both sterile recovery bees and bees exposed to untreated hive workers (Chi-squared test, *p* < 0.0001) (S2B and S2C Fig). In contrast, for germ-free bees, antibiotic treatment did not result in increased mortality compared to controls (S2D Fig).

Bees were sampled on Days 3, 5, and 7 to evaluate the long-term effects of antibiotic exposure on gut community composition. Bees treated with tetracycline and returned to the hive displayed changes in both community composition and size at all post-treatment sampling points (Wilcoxon test, *p* < 0.05) (Fig 2B–2D). Control bees had, on average, five times more bacterial cells in their guts than bees treated with tetracycline, and this discrepancy was evident at all post-treatment sampling time-points (Fig 2B and 2C). The four core taxa that decreased in absolute abundance at Day 0 (Wilcoxon test, *p* < 0.001) (i.e., *Bifidobacterium*, Firm-4, Firm-5, and *S*. *alvi*) continued to be significantly decreased at all time-points (S3A–S3D Fig). Another core species, *Bartonella apis*, which was not significantly altered at Day 0, was decreased at all subsequent sampling time-points (Wilcoxon test, *p* < 0.001) (S3E Fig). Additionally, the absolute abundance of a few other species shifted at various time-points after the bees were returned to the hive (Wilcoxon test, *p* < 0.05). Two core species (Alpha 2.1 and *Frischella perrara*) and one environmental species (*Lactobacillus kunkeei*) were decreased at one or more time-points (S4A–S4C Fig). In contrast, several non–core taxa, including the genus *Serratia* and unclassified bacteria in the family *Halomonadaceae*, showed elevated abundance at Days 3 and 5, respectively (Wilcoxon test, *p* < 0.05) (S4D and S4E Fig). Complementary experiments in which bees were kept in the lab after tetracycline treatment exhibited similar effects on community size and composition (S5 Fig), but did not show an increase in non—core bacteria.

In addition to an increase in non-core bacterial taxa in tetracycline-treated bees, we also observed an apparent increase in fungal sequences in treated bees at Days 3–7, based on diagnostic PCR assays (S6A Fig). However, identified fungal taxa (see Materials and methods) were all closely related to yeast taxa isolated from flowers (S6B Fig), suggesting that these are transient in guts of these bees, and are likely more abundant and detectable in treated bees because fewer bacteria are present. In some treated bees, the typically specific fungal primers amplified plant DNA (S6B Fig), suggesting that fungal DNA template is rare in the samples.

After bees were returned to the hive, differences in community composition were also apparent in the relative abundances of individual species (Fig 2D and S7 and S8 Figs). The mean relative abundances of bacterial species remained stable in control bees over all sampling periods, whereas treatment bees displayed a major shift in gut microbial composition that was not stable over time and did not return to the baseline composition after one week (S7 Fig). Concerning the core bacteria of the gut, the relative abundances of *Bifidobacterium*, Firm-4, Firm-5, and *B*. *apis* were decreased at Days 3, 5, and 7 (S7 and S8 Figs). However, the relative abundance of *Gilliamella apicola* was much higher in treated bees (S7 and S8 Figs).

**Fig 3:**
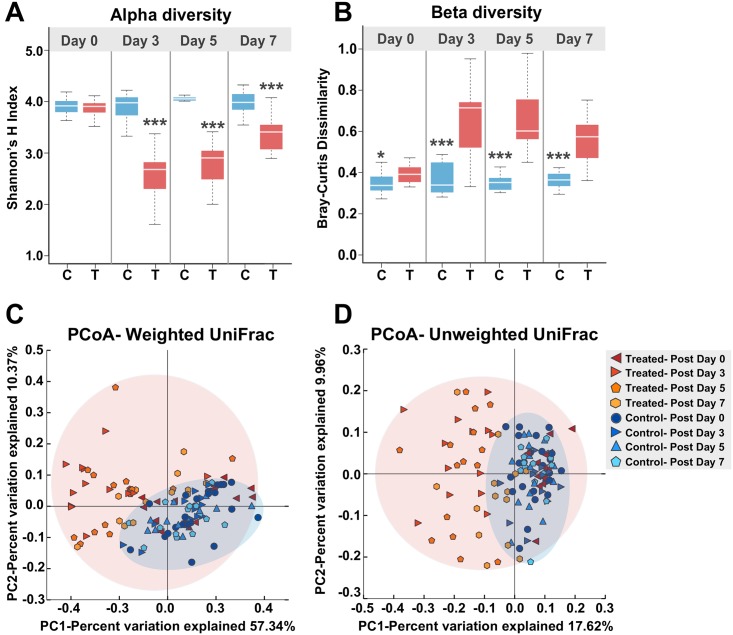
Alpha and beta diversity of treatment and control bees. **A)** Difference in alpha diversity between control and treatment bees at each time-point (measured as Shannon’s H). **B)** The average Bray-Curtis dissimilarity in gut communities among control bees versus between control bees and treatment bees. Box-and-whisker plots show high, low, and median values, with lower and upper edges of each box denoting first and third quartiles, respectively. * = *p* < 0.05 and *** = *p* < 0.0001, Wilcoxon rank sum tests. **C)** Principal coordinate analysis using unweighted UniFrac. **D)** Principal coordinate analysis using weighted UniFrac. See S3 Data for alpha and beta diversity data.

Based on relative abundance, antibiotic treatment also caused changes in microbiota diversity within individual hosts (alpha diversity) and in microbiota divergence between individual hosts (beta diversity) (Wilcoxon test, *p* < 0.05) (Fig 3). Alpha diversity, measured as Shannon’s H index, was lower in treatment bees at all time points except Day 0 (Wilcoxon test, *p* < 0.0001) (Fig 3A). Beta diversity, measured as the average Bray-Curtis dissimilarity, was lower among control bees than between control and treatment bees at all time-points (Wilcoxon test *p* < 0.0001) (Fig 3B). Principal coordinate analysis (unweighted and weighted UniFrac, [29]) showed that gut community compositions of treatment bees are widely dispersed in contrast to the tight clustering observed for control bees (Fig 3C and 3D). Furthermore, for the bees retained in laboratory cages, the gut community compositions of treatment versus control bees displayed similar clustering patterns based on unweighted and weighted UniFrac: treated bees had more dispersed communities for both sterile bees and bees exposed to other bees in social groups (S9A–S9D Fig).

**Fig 4:**
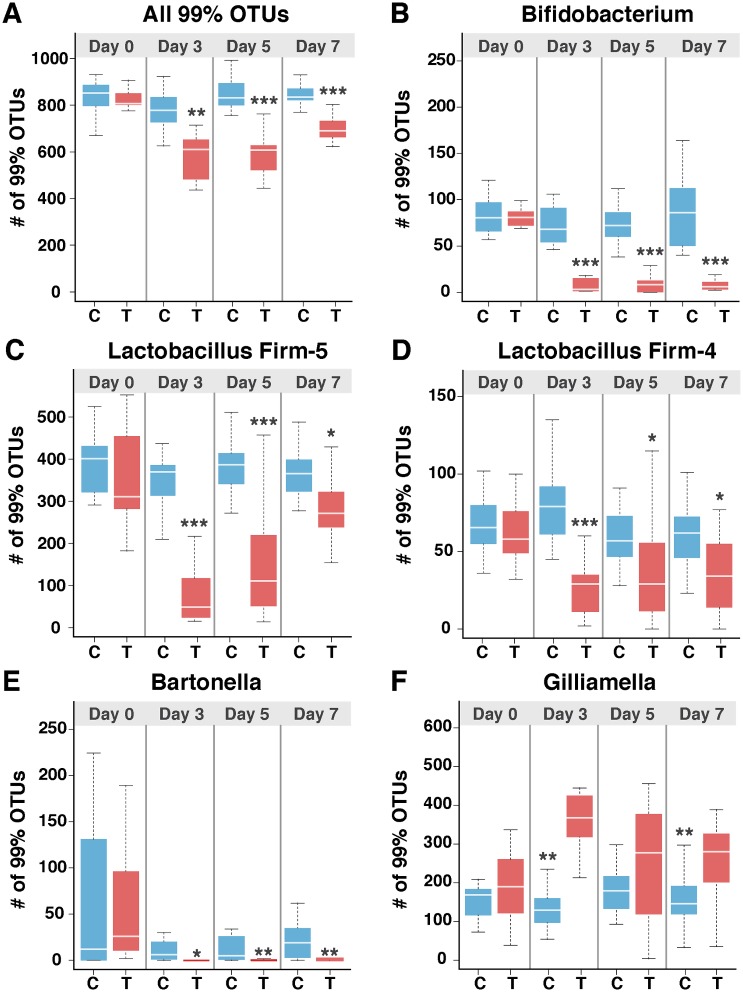
Changes in fine-scale bacterial diversity in tetracycline-treated bees based on 99% OTUs detected at Days 0, 3, 5, and 7 post-treatment. A) Total numbers of bacterial 99% OTUs present in control and treatment bees B-E) Numbers of 99% OTUs for the four species (*Bifidobacterium*, Firm-5, Firm-4, and *B*. *apis*) that displayed a significant decrease in diversity at Days 3, 5, and 7 post-treatment. F) Numbers of OTUs for the genus *Gilliamella*, which showed significant increases in OTU diversity at Days 3 and 7 in treatment bees. Box-and-whisker plots show high, low, and median values, with lower and upper edges of each box denoting first and third quartiles, respectively. * = *p* < 0.05, ** = *p* < 0.001 and *** = *p* < 0.0001, Wilcoxon rank sum tests. See S4 Data for 99% OTU data.

To evaluate the effects of tetracycline treatment on fine-scale strain and species diversity, we counted the number of 99% operational taxonomic units (OTUs) assigned to each genus. At Day 0 post-treatment, no significant differences were seen in 99% OTU diversity, but at Days 3, 5, and 7, the total number of OTUs was significantly lower in treatment bees (Wilcoxon test, *p* < 0.05) (Fig 4A). In particular, *Bifidobacterium*, Firm-4, Firm-5, and *B*. *apis* showed a decrease in fine-scale diversity (Wilcoxon test, *p* < 0.05) (Fig 4B–4E). This is consistent with the decrease in relative and absolute abundance of these species (Fig 1 and S3 Fig). Furthermore, the increase in relative abundance of *G*. *apicola* also corresponded to an increase in 99% OTU diversity at Days 3 and 7 post-treatment (Wilcoxon test, *p* < 0.001) (Fig 4F).

**Fig 5:**
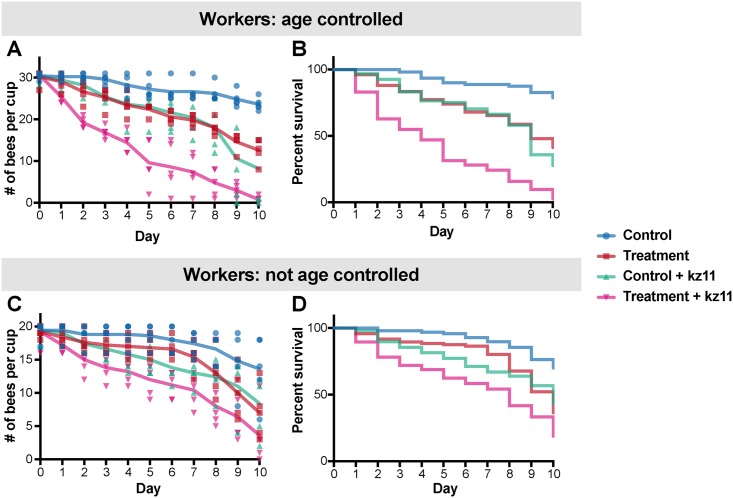
Survivorship of honeybees exposed to *Serratia* after tetracycline treatment. **A)** Number of control and tetracycline-treated honeybees (age-controlled) alive after exposure to *Serratia* kz11. Five replicates were performed for each group. Control bees were fed sterile sugar syrup for 5 d, and treatment bees were administered 450 ug/ml of tetracycline in sterile sugar syrup for 5 d, followed by exposure to i) *Serratia* kz11 or ii) sterile sugar syrup only. Survivorship was monitored and recorded each day for 10 d (S7 Data). **B)** The percent survival of age-controlled bees after *Serratia* exposure, shown as a Kaplan—Meier survival curve created using GraphPad Prism. Statistical analyses were performed using the coxph model implemented in the “survival” package [30] in R (S5 Data). **C)** Number of control and tetracycline-treated honeybees (non-age-controlled) alive after exposure to *Serratia* kz11. Five replicates were performed for each group. Survivorship was monitored and recorded each day for 10 d (S8 Data). **D**) Kaplan—Meier survival curve showing the percent survival of non-age-controlled bees after *Serratia* exposure. The survival curve was created using GraphPad Prism. Statistical analyses were performed using the coxph model implemented in the “survival” package [30] in R (S6 Data).

In order to investigate whether opportunistic pathogens contribute to the increased mortality observed for treated bees returned to the hive, we performed infection experiments using a *Serratia* strain that was isolated from honeybee guts (*Serratia* kz11). In one experiment, we exposed age-controlled bees (emerged in the lab on the same day) to *Serratia* kz11 after treatment with tetracycline (see Materials and methods). In the other experiment, we exposed non-age-controlled (bees taken from a brood frame in the hive). In both experiments, the bees were exposed to *Serratia* kz11 through their food for 2 d. (Viable *Serratia* cells can be obtained from bee bread up to 2 d after inoculation). In both age-controlled and non-age-controlled experiments, bees treated with tetracycline and exposed to *Serratia* kz11 exhibited increased mortality when compared to control bees, bees exposed to tetracycline only, or bees exposed to *Serratia* only (Fig 5, S5 Data and S6 Data).

**Fig 6:**
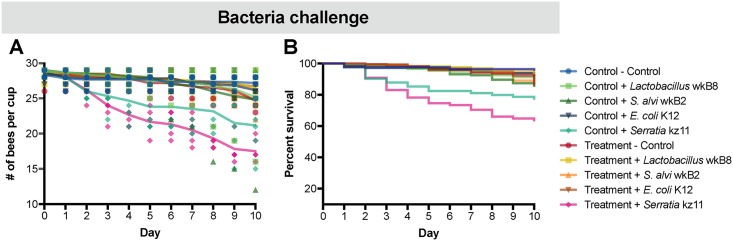
Bacterial challenge experiment. A) Number of control and tetracycline honeybees alive after exposure to different bacterial strains. Control bees were fed sterile sugar syrup for 5 d, and treatment bees were administered 450 ug/ml of tetracycline in sterile sugar syrup for 5 d, followed by exposure to i) *Serratia* kz11, ii) *E*. *coli* K-12, iii) *S*. *alvi* wkB2, iv) *Lactobacillus* sp. wkB8, or v) no bacteria. Five replicates were performed for each group. Survivorship was monitored and recorded each day for 10 d (S10 Data). **B)** Kaplan—Meier survival curve showing the percent survival of control and tetracycline-treated bees after bacteria exposure. The survival curve was created using GraphPad Prism. Statistical analyses were performed using the coxph model implemented in the “survival” package [30] in R (S9 Data).

To confirm that *Serratia* kz11 can be an opportunistic pathogen of honeybees, we performed a bacterial challenge experiment in which we exposed bees to different bacteria following tetracycline treatment. We exposed control and treatment bees to i) *Serratia* kz11, ii) *Escherichia coli* K-12, iii) *S*. *alvi* wkB2, iv) *Lactobacillus* sp. wkB8. The latter two species are part of the core bee gut microbiome [12], and *E*. *coli* K-12 is a nonpathogenic lab strain [31]. Only bees exposed to *Serratia* kz11 showed an increase in mortality in control and treated bees when compared to control bees (Fig 6, S9 Data).

## Discussion

Since gut community members engage in mutualistic interactions, such as cross-feeding, and antagonistic interactions, such as competition and direct killing, responses of different species and genotypes to environmental changes are interdependent. These interactions can be altered or eliminated as a consequence of antibiotic treatment, potentially impacting host health. Several studies have shown that the use of antibiotics causes alterations in the microbiomes of humans and livestock (reviewed in [32]). In honeybees and bumblebees, globally important pollinators, gut communities have been implicated in both nutrition and defense against pathogens [19–22]. In relation to growing evidence for the importance of the gut microbiome in animal health [32] and the largely unexplained decline of honeybee colonies [18], the effects of antibiotic treatment on the honeybee gut microbiome are of major interest.

Tetracycline, a broad-spectrum antibiotic, targets both Gram-positive and Gram-negative bacteria and is thus expected to affect multiple members of the gut community. As predicted, we observed substantial changes in the gut microbial community composition and size following treatment with tetracycline. However, none of the core bacterial species was completely eliminated. This persistence may have been enhanced by the presence of antibiotic resistance. In US honeybees, core species of the microbiota carry tetracycline resistance genes, which persist at low frequencies, even in hives with no recent history of antibiotic treatment [27]. Thus, we expect some tetracycline resistance in our hives, which had not been treated for over 2 y prior to our study. Nevertheless, most core species declined in population size and/or diversity following treatment. An exception was *G*. *apicola*, for which relative abundance as well as strain-level diversity increased after treatment. Overall, the effects of tetracycline treatment on the gut microbiome composition were more prominent several days after treatment was stopped, probably due to a delayed effect of the antibiotics. Also, dead bacterial cells may have accumulated in the gut from lack of defecation while bees were maintained in laboratory cages, causing our DNA-based profiles to fail to reveal initial declines in numbers of living cells. Even so, significant declines in the overall community size were seen for all treatment bees starting on the first day of sampling. Moreover, the same delayed effect was observed in treated bees kept in the lab throughout the recovery period.

We found that honeybees treated with antibiotics and returned to the hive had decreased survivorship when compared to untreated bees. Several studies have pointed to a role for the bee gut microbiome in protection against trypanosomatid pathogens [19,21,33]. One recent study showed that the colonization order of honeybee gut symbionts affects susceptibility to infection by the pathogenic trypanosomatid *Lotmaria passim* [21], providing evidence that gut dysbiosis promotes pathogen invasion. We detected elevated levels of two groups of non—core bacteria, *Serratia* and an unclassified *Halomonadaceae*, in treated bees sampled from the hive; these could represent opportunistic pathogens able to invade the gut as a result of antibiotic perturbation. Members of the family *Halomonadaceae* generally inhabit high-saline and pH environments, but some have been recognized as human pathogens [34,35]. *Halomonadaceae*-related taxa have been detected in microbiome studies of the honeybee gut and pollen, but their status in bees is unknown [36]. *Serratia* is an opportunistic pathogen in humans and many animals, including insects [37,38]. Along with other Enterobacteriaceae, it is widely present at low frequencies in honeybee guts where it is considered a signifier of atypical microbiome composition in bees [15,39]. Therefore, one or both of these bacteria could be responsible for the increased morality in treated bees. To test this, we exposed treated and control bees to a *Serratia* strain isolated from honeybees. We observed that *Serratia* exposure resulted in elevated mortality in bees that had been treated with tetracycline. Furthermore, this *Serratia* strain shows relatively high resistance to tetracycline (see Materials and methods for details), suggesting that it would also have a selective advantage during the course of antibiotic treatment.

Dysbiosis can lead to the sudden overgrowth and pathogenic behavior of opportunistic organisms (pathobionts) already present in the gut [40]. Perturbations of the gut community can affect gene expression, protein activity, and the overall metabolism of the gut microbiota [41]. For example, changes in microbial community structure can alter the provision of nutrients or secondary metabolites and inhibit the removal of toxic metabolites [42]. Metagenomic analysis of colonies with colony collapse disorder (CCD), showed increases in relative abundances of *G*. *apicola*, *F*. *perrara*, *S*. *alvi*, and *Lactobacillus* and decreases in *Alphaproteobacteria* and *Bifidobacteria* when compared to healthy hives [43]. We also observed an increase in the abundance and diversity of *G*. *apicola* in treated bees within hives. These results suggest negative effects of high abundance of *G*. *apicola* or of reduced abundances of *Bifidobacterium* and *Lactobacillus*, which are thought to be protective in humans and other animals, including honeybees [44–46]. Furthermore, bees with naturally acquired microbiomes that were treated with antibiotics and kept in sterile conditions in the lab exhibited increased mortality rates, which were not observed in treated bees lacking their microbiome (germ-free bees), implying that dysbiosis alone, rather than the tetracycline treatment itself, can impact bee health. Although the lack of an effect on germ-free bees suggests that the antibiotic is not directly harmful to bees in the concentrations we used, it is difficult to disentangle effects of tetracycline on the gut microbiome from effects on the host, which may in turn alter susceptibility to pathogens.

Antibiotics are commonly used in apiculture in several countries [47] and are administered to hives by mixing with powdered sugar, sugar syrup, or dietary extender patties. The recommended treatment involves feeding or dusting each hive with approximately 200 mg/oz of tetracycline three times in the spring and fall at intervals of 4–5 d. The dose we administered in this study was slightly lower than that used in apiculture. In actual hive conditions, it is unclear how much antibiotic individual bees would consume, but it is likely that some bees receive doses as high or higher than those used here. Additionally, when antibiotics are administered to hives, they can persist for long periods of time. For example, tetracycline has been detected in treated hives for up to 3 mo post-treatment [47,48]. Therefore, the effects on treated hives could be greater than what we report here. Our results suggest that treated bees allowed to recover in the hive without further exposure revert towards their baseline microbiome composition after 1 wk (Fig 3). Potentially, negative effects of antibiotic treatment could be reduced through the development of alternative treatment methods that allow for the removal of the antibiotic from the hive after a specified period.

In this study, we found that tetracycline, a commonly used antibiotic in beekeeping and in other livestock, severely alters the gut microbiome composition of honeybees and decreases survivorship within hives. These results thus suggest a beneficial role of the normal gut microbiome in honeybee health within the hive environment. A possible implication is that the use of antibiotics in beekeeping can be detrimental because of interference with these benefits. We show that a strain of *Serratia* isolated from bees causes increased mortality following tetracycline treatment. Antibiotic-induced dysbiosis may also lead to increases in nonbacterial pathogens, such as viruses and eukaryotes, as shown for trypanosomatids [19,21]. Other consequences of dysbiosis, such as nutritional impacts or heightened susceptibility to toxins, may also contribute to the survivorship decline that we observed in antibiotic-treated bees. Although our results suggest that antibiotic treatments may be detrimental for honeybees, we emphasize that many other factors contribute to pollinator declines, which are affecting many wild pollinator populations that are not exposed to antibiotics [49]. Therefore, antibiotic treatment can only be considered a single potential factor amongst a myriad of other potential culprits, including loss of foraging habitat. Furthermore, antibiotic treatments sometimes may be highly beneficial, to prevent or control infections by foulbrood agents [23].

Overall, our findings underline the usefulness of honeybees as a model system for disentangling the fine-scale dynamics of perturbed gut communities. Furthermore, the honeybee gut system can provide fundamental information on how antibiotic treatment affects the normal microbiota and host health.

## Materials and methods

### Hive recovery experiment

Approximately 800 adult worker bees were collected from brood frames from a single hive (Big Top) kept at the University of Texas in Austin (UT). The bees were immobilized at 4°C and marked with green or pink Testors paint. The bees were separated into two groups, control and treatment, green and pink, respectively, and were distributed to cup cages of previously described design [50]. The cup cages were maintained in growth chambers at 35°C and 90% relative humidity to simulate hive conditions. Each cup cage contained 30 bees, with 15 replicates for each condition. Control bees were fed filter-sterilized 0.5 M sucrose syrup and treatment bees were fed 450 ug/ml of tetracycline suspended in filter-sterilized 0.5 M sucrose syrup. After 5 d, 15 bees were sampled (one from each cup cage), placed in 100% ethanol, and stored at 4°C. The remaining bees (345 control and 340 treatment) were then returned to their original hive. Three days after reintroduction to the hive, the bees were individually captured and temporarily kept in cup cages (40 bees per cup) until all marked bees had been recovered from the hive. After counts were obtained, 15 marked bees for both control and treatment groups were collected from each hive at time-points of 3, 5, and 7 d following antibiotic treatment (Nov. 6–13, 2015), placed in 100% ethanol and stored at 4°C. Within 2 wk of collection, the bees were removed from cold ethanol and the entire gut from crop to rectum was homogenized and placed into a bead-beating tube in 500 uL of 100% molecular grade ethanol and stored at −20°C. Dissections were performed with flame-sterilized forceps under aseptic conditions. DNA was extracted from the gut using established techniques, Illumina-based amplicon profiling of the V4 region of 16S rRNA gene was performed (S11 Data), and the community size pre- and post-treatment was quantified by qPCR using the total number 16S rRNA gene copies, adjusting for number of rRNA operons per genome (S1 Table).

A replicate hive survival experiment was performed using a different hive (Chickamauga) kept at UT. Approximately 1,300 adult worker bees were collected from brood frames from a single hive (Nov. 1, 2016). The bees were immobilized at 4°C and marked with green or pink Testors paint. The bees were separated into two groups, control and treatment, pink and green, respectively, and were distributed to cup cages. The cup cages were maintained in growth chambers at 35°C and 90% relative humidity to simulate hive conditions. Each cup cage contained 30 bees, with 22 replicates for each condition. Control bees were fed filter-sterilized 0.5 M sucrose syrup, and treatment bees were fed 450 ug/ml of tetracycline suspended in filter-sterilized 0.5 M sucrose syrup. After 5 d, the remaining bees (622 control and 623 treatment) were returned to their original hive. After reintroduction to the hive, post-treatment survival in the hive was assessed by counting bees on d 3. We individually captured bees and temporarily kept them in cup cages (40 per cup) until all marked bees had been recovered.

### In laboratory recovery experiment

A single brood frame was removed from a hive (Big Top) at the UT campus and was placed in a growth chamber at 35°C and 90% humidity. Pupae were allowed to emerge naturally, and newly emerged adults (NEWs) were collected after 24 h. Approximately 600 NEWs were marked with yellow Testors paint and returned to their original hive in order to naturally acquire their microbiota. After 7 d in the hive, approximately 450 bees were recaptured. They were then briefly immobilized at 4°C and split into two groups, control and treatment. These bees were placed in cup cages and maintained in growth chambers. Each cup cage contained approximately 22 bees (ten replicates for each condition). Control bees were fed filter-sterilized 0.5 M sucrose syrup, and treatment bees were fed 450 ug/ml of tetracycline suspended in filter-sterilized 0.5 M sucrose syrup. Any dead bees were removed during a daily census. After 5 d, ten bees were sampled from each treatment group (one from each cup cage), placed in 100% ethanol, and stored at 4°C, and the remaining bees were chilled and randomly redistributed into new cup cages. The bees were then allowed to “recover” under the following conditions i) exposed, i.e., with normal workers collected from their hive (15 per cup cage) or ii) sterile, i.e., kept only with other tetracycline-treated bees from their cohort. Ten bees were collected from each of the four groups (i) control exposed, ii) control sterile, iii) treatment exposed, iv) treatment sterile at time-points of 0, 3, 5, and 7 d following antibiotic treatment (Nov. 3–10, 2015) placed in 100% ethanol and stored at 4°C. Within 2 wk of collection, the bees were removed from cold ethanol, and the entire gut from crop to rectum was homogenized and placed into a bead-beating tube in 500 uL of 100% molecular grade ethanol and stored at −20°C. Dissections were performed with flame-sterilized forceps under aseptic conditions. DNA was extracted from the guts (see below), Illumina-based amplicon profiling of 16S rRNA gene copies was performed, and the total community size pre- and post-treatment was quantified by qPCR using the number of 16S rRNA gene copies (adjusting for number of rRNA operons per genome). In order to compare the differences in survivorship effects in the hive and in the lab, the survivorship of the lab-kept bees was also measured on d 3 post-treatment.

### Germ-free survival assay

A brood frame was collected from a hive at UT (Blueberry). Late-stage pupae (eyes pigmented but pupae lacking movement) were removed from these frames and placed on cotton pads in sterile plastic bins. These were placed in growth chambers at 35°C and high humidity (∼90% relative humidity) to simulate hive conditions, and pupae were allowed to eclose naturally. After eclosure, NEWs were briefly immobilized at 4°C, and bins were combined to randomize potential age variation. The NEWs were then placed in cup cages and maintained in growth chambers. Each cup cage contained approximately 13 bees (four replicate cups for each condition). Control bees were fed filter-sterilized 0.5 M sucrose syrup and sterile bee bread, and treatment bees were fed 450 ug/ml of tetracycline suspended in filter-sterilized 0.5 M sucrose syrup and sterile bee bread. Any dead bees were removed during a daily census. After 5 d, tetracycline treatment was arrested, and all bees were fed filter-sterilized 0.5 M sucrose syrup and sterile bee bread. Survivorship of the germ-free bees was measured on d 3 post-treatment by counting the total number of bees alive for each group (controls and treatments).

### DNA extraction

To extract DNA from bee tissues, we used bead-beating with cetyltrimethylammonium bromide (CTAB), method as described in [11] with the following modifications: after bead-beating, the samples were allowed to sit briefly at 56°C while the foam settled. We then added 1 ul RNase A solution (Sigma) and vortexed the tubes briefly and left them overnight at 56°C. We then added 0.75 ml phenol-chloroform-isoamyl alcohol (25:24:1) (Ambion, Austin, TX, USA) to each tube, shook for 30 s before placing on ice for at least 2 min, and then centrifuged at full speed for 30 min at 4°C. The aqueous phase was precipitated in alcohol, washed, and air-dried, then resuspended in 50 ul nuclease-free water.

### Quantative PCR to estimate bacterial abundance

We amplified total copies of the 16S rRNA gene using universal bacterial primers with an Eppendorf Mastercycler ep realplex instrument (Eppendorf, Hauppauge, NY, USA). The forward primer was 27F (5’-AGAGTTTGATCCTGGCTCAG-3’), and the reverse primer was 355R (5’-CTGCTGCCTCCCGTAGGAGT-3’). Reactions (10 ul) were carried out in triplicate with 5 ul iTaq universal SYBR Green (Bio-Rad, Inc.), 1 ul (each) 3 uM primer, 2 ul H_2_O, and 1 ul of template DNA that had been diluted 100X. The PCR cycle was 95°C (3 min) followed by 40 cycles of 95°C (3 s) and 60°C (20 s). Using standard curves from amplification of the cloned target sequence in a pGEM-T vector (Promega, Madison, WI, US), we calculated absolute copy number for the reaction template then adjusted this based on dilution to calculate the total copy number for each sample.

### Illumina sequencing

PCR amplifications of the V4 region of the 16S rRNA gene were performed in triplicate using 515F and 806R primers, as described previously [51]. Reaction products were purified with AMPure XP Beads (Beckman Coulter). The resulting amplicons were subjected to Illumina sequencing on the MiSeq platform (2x250 sequencing run) at the Genome Sequencing and Analysis Facility at UT.

### Sequence analysis

Illumina sequence reads were processed in QIIME [52]. FASTQ files were filtered for quality with split_libraries_fastq.py allowing a minimum Phred quality score of Q20. Forward and reverse Illumina reads were joined using join_paired_ends.py with default settings. Chimeric sequences were removed using the usearch6.1 detection method implemented in the identify_chimeric_seqs.py script in QIIME. OTUs were clustered at 97% and 99% using the UCLUST algorithm as implemented in pick_open_reference_otus.py. Briefly, sequence reads were initially clustered against the July 2015 release of the SILVA [53] reference data set (http://www.arb-silva.de/download/arb-files). Sequences that did not match the SILVA data set were subsequently clustered into de novo OTUs with UCLUST. Unassigned, mitochondrial, and chloroplast reads were removed from the dataset. To eliminate pyrosequencing errors all OTUs present in less than 0.1% abundance were removed. Because the currently available curated 16S rRNA sequence databases do not contain reference sequences for the core species of the honeybee gut microbiota, taxonomic assignment was performed using a local BLAST database of reference honeybee bacteria 16S rRNA gene sequences.

Downstream analyses including alpha and beta diversity estimations were conducted using the QIIME workflow core_diversity_analysis.py, with a sampling depth of 5,000 reads per sample and default parameters. Rarefaction depths were chosen manually to exclude samples with exceptionally low total sequences (see S11 Data for sample details). The absolute abundance of each bacterial species was estimated by multiplying the total number of 16S rRNA genes (measured by qPCR and adjusting for rRNA operons per genome) by the percent relative abundance of each species. The number of 16S rRNA operons was determined using the reference genome for a given bee gut bacterial species when available. When complete genomes were not available, the mean 16S rRNA operon copy number for the bacterial genus or family was obtained from the rrnDB (https://rrndb.umms.med.umich.edu/) and used as an estimation of the copy number (S1 Table). Statistical tests were performed using the Wilcoxon rank sum test and the Chi-squared test implemented in R.

### Screen for fungi

The hive (Big Top) recovery bees (all treatment and control bees from d 0, 3, 5, and 7) were screened for the presence of Fungi using diagnostic PCR with the universal fungal primers ITS1-F (5'-CTTGGTCATTTAGAGGAAGTAA-3') and LR3- R (5'-GGTCCGTGTTTCAAGAC-'3) [54,55]. PCR assays included positive (purified *Saccharomyces cerevisiae* DNA) and negative (ddH_2_O) controls. PCR amplification of DNA products was performed using the Taq Polymerase (TaKaRa, Japan). PCR amplification was carried out as follows: 94°C for 4 min and 40 cycles of 30 s at 94°C, 30 s at 55°C, and 1 min at 72°C; and 72°C for 10 min. After completion of the PCR, 5 μL of the samples was electrophoresed on a 2% agarose gel to determine whether the DNA of interest was amplified. The amplified products were then purified using AMPure XP Beads (Beckman Coulter) and cloned using the pGEM-T Easy Vector Systems (Promega, Madison, WI, US) according to the manufacturers instructions. The recombinant plasmids were transformed into the competent *E*. *coli* DH5α. The transformation was plated on LB Agar containing 100 mg/l each of ampicillin, IPTG and X-gal. White colonies were screened using PCR with plasmid insert specific primers T7 (5´-TAATACGACTCACTATAGGG-3´) and SP6 (5´-ATTTAGGTGACACTATAG-3´). PCR amplification was carried out as follows: 95°C for 2 min. and 28 cycles of 10 sec. at 95°C, 20 sec. at 46°C, and 90 sec. at 68°C; and 68°C for 1 min. A total of 20 clones were selected randomly, and inserts were sequenced at both ends, using Sanger sequencing services at the DNA Sequencing Facility (DSF) at UT. After trimming, 34 high quality end sequences were retained. These end-reads were used as queries in BLASTn searches against the NCBI non-redundant nucleotide database.

### Isolation of *Serratia* from honeybee gut microbiome

A honeybee package was purchased from a commercial apiary that does not treat with antibiotics or other chemicals. The package of workers was kept in the lab for approximately one month and was provided with 0.5 M sucrose solution. Hundreds of honeybees were dissected at different time-points, homogenized, and preserved in 20% glycerol at −80°C. Homogenized guts were then plated onto heart infusion agar plates with sheep’s blood and placed at 37°C in a 5% CO_2_ chamber overnight. Single bacterial colonies were isolated and stored in 20% glycerol at -80°C. Purified isolates were then amplified using PCR with universal bacterial primers 27F and 1492R [56] and Sanger-sequenced at the DNA Sequencing Facility (DSF) at UT. Sequences were identified by BLAST searches against the nonredundant nucleotide database of NCBI. One *Serratia* strain was chosen as a potential pathogen for infection experiments (kz11). *Serratia* kz11 was tested for tetracycline resistance based on the Minimum Inhibitory Concentration (MIC) technique using 0.016–256 μg/mL E-test strips (bioMérieux). In brief, *Serratia* kz11 was plated onto LB plates. When the surface of each plate had dried, one E-test strip was put on each plate. The plates were incubated with the lid-side up at 37°C for approximately 24 h. MICs were read directly from the test strip according to the instructions of the manufacturer, where the elliptical zone of inhibition intersected with the MIC scale on the strip. The MIC was >32 μg/ml for *Serratia* kz11. We note that nonresistant *Serratia* strains are known, with MIC < 1 [57].

### *Serratia* infection experiment on age-controlled workers

A single brood frame was removed from a hive (Whiskeytown) at the UT campus and was placed in a growth chamber at 35°C and 90% humidity. Pupae were allowed to emerge naturally, and NEWs were collected after 24 h. A total of 720 NEWs emerged and were distributed into cup cages and fed with freshly prepared worker hindgut homogenate in addition to their food, which allows the NEWs to acquire their core microbiome composition [11]. Each cup cage contained approximately 45 bees (16 replicate cups). After 6 d, the bees were immobilized at 4°C, separated into cohorts (control and treatment), and distributed to cup cages. The cup cages were maintained as described above in growth chambers simulating hive conditions. Each cup cage contained approximately 43 bees, with eight replicates for each condition. Control bees were fed filter-sterilized 0.5 M sucrose syrup and treatment bees were fed 450 ug/ml of tetracycline suspended in filter-sterilized 0.5 M sucrose syrup. After 5 d, the tetracycline treatment was stopped. One day later, the bees were chilled, randomly redistributed into new cup cages, and exposed to *Serratia* kz11 or fed only sterile sugar syrup and sterile bee bread. This resulted in a total of four groups with approximately 150 bees (five replicate cups with approximately 30 bees per cup) in each group: 1) control (no *Serratia*), 2) control + *Serratia* kz11, 3) post-treatment (no *Serratia*), 4) post-treatment + *Serratia* kz11. Bees were censused every day for ten d following bacterial exposure. Kaplan—Meier survival curves were generated in GraphPad Prism version 7.0b for Mac OS X, GraphPad Software, La Jolla, California USA, www.graphpad.com. Statistical analyses were performed in R using the Cox proportional hazard model (coxhp) implemented in the “survival” package [30].

### Serratia infection experiment on non-age-controlled workers

Approximately 600 adult worker bees were collected from a brood frame from a hive (Big Top) kept at UT (Jul. 21, 2016). The bees were immobilized at 4°C, separated into cohorts (control and treatment), and distributed to cup cages of previously described design [50]. The cup cages were maintained as described above in growth chambers simulating hive conditions. Each cup cage contained 20 bees, with 15 replicates for each condition. Control bees were fed filter-sterilized 0.5 M sucrose syrup and treatment bees were fed 450 ug/ml of tetracycline suspended in filter-sterilized 0.5 M sucrose syrup. After 5 d, the tetracycline treatment was stopped. One day after tetracycline treatment was arrested the bees were chilled, randomly redistributed into new cup cages, and exposed to *Serratia* kz11 isolated from honeybee guts or were fed only sterile sugar syrup and sterile bee bread. This resulted in a total of four groups with 100 bees (five replicate cups with approximately 20 bees per cup) in each group: 1) control (no *Serratia*), 2) control + *Serratia* kz11, 3) post-treatment (no *Serratia*), and 4) post-treatment + *Serratia* kz11. Censusing and statistical analyses were the same as for the experiment on age-controlled bees, described above.

### Bacterial challenge experiment

Approximately 1,800 adult worker bees were collected from a brood frame from a hive (Whiskeytown) kept at the UT campus (Nov. 14, 2016). The bees were immobilized at 4°C, separated into cohorts (control and treatment), and distributed to cup cages. The cup cages were maintained as described above in growth chambers simulating hive conditions. Each cup cage contained 45 bees, with 20 replicates for each condition. Control bees were fed filter-sterilized 0.5 M sucrose syrup and treatment bees were fed 450 ug/ml of tetracycline suspended in filter-sterilized 0.5 M sucrose syrup. After 5 D, the tetracycline treatment was stopped. One day after tetracycline treatment was arrested (Day 1), the bees were chilled, randomly redistributed into new cup cages, and exposed to i) *S*. *alvi* wkB2, ii) *Lactobacillus* sp. wkB8, iii) *E*. *coli* K-12, iv) *Serratia* kz11, or v) no bacteria (controls). This resulted in a total of ten groups with approximately 170 bees (six replicate cups with approximately 28 bees per cup) in each group: 1) control (no *Serratia*), 2) control + *Serratia* kz11, 3) control + *Lactobacillus* sp. wkB8, 4) control + *S*. *alvi*, 5) control + *E*. *coli*, 6) post-treatment (no *Serratia*), 7) post-treatment + *Serratia* kz11, 8) post-treatment + *Lactobacillus* sp. wkB8, 9) post-treatment + *S*. *alvi*, and 10) post-treatment + *E*. *coli*. Censusing and statistical analyses were the same as for the experiment on age-controlled bees, described above.

### Bacterial culturing and administration

*Serratia* kz11 and *E*. *coli* K-12 were grown in liquid LB media at 37°C overnight. *S*. *alvi* wkB2 was grown in Insectagro (Corning) for 3 D at 37°C in 5% CO_2_. *Lactobacillus* sp. wkB8 was grown in MRS broth for 3 D at 37°C in 5% CO_2_. The 600 nm optical densities for each bacterial culture were measured, and cells were washed three times with PBS and diluted to a concentration of 0.5 OD in sterile sugar syrup or in PBS. The bacteria—PBS solutions were applied to sterile bee bread (gamma-irradiated) in feeding troughs that were placed in the cup cages and the bacteria—sugar syrup solutions were administered in feeding vials on Day 1 post—tetracycline treatment.

In order to determine how long viable bacterial cells remained on the bee bread, feeding troughs (ten per bacterial treatment) were filled with sterile bee bread and inoculated with a concentration of 0.5 OD of bacteria suspended in PBS. Control bee bread was inoculated with PBS only. Each day, one trough was sampled, mixed with 500 ul of PBS and plated out in triplicate (LB agar for *Serratia* kz11 and *E*. *coli* K-12, heart infusion agar (Difco) supplemented with 5% sheep’s blood for *S*. *alvi* wkB2, MRS agar for *Lactobacillus* sp. wkB8, and one of each for controls). The plates were checked for bacterial growth after 24 h at 37°C (*Serratia* and *E*. *coli*) or 72 h at 37°C in 5% CO_2_ (*S*. *alvi* and *Lactobacillus*). Viable cells were detected for i) *Serratia* kz11 up to Day 2, ii) *Lactobacillus* sp. wkB8 up to Day 3, iii) *S*. *alvi* wkB2 up to Day 3, and iv) *E*. *coli* K-12 up to Day 4. Viable cells were never isolated from control (l) bee bread.

### Nucleotide sequence accession number

16S rRNA gene reads are deposited with NCBI Sequence Read Bioproject: PRJNA338694.

## Supporting information

S1 Fig Effects of tetracycline treatment on relative abundances of the eight core gut bacteria. A) Stacked column graphs showing the relative abundances of bee gut bacterial species in control bees (n = 14) and treatment bees (n = 15) after five days of tetracycline treatment (Day 0 post-treatment), see Dataset S7 for sample details. B) Boxplots showing the relative abundances of the eight core bee gut species in control and treatment bees on Day 0. None of the eight core species showed significant changes in relative abundance following tetracycline exposure at Day 0 (NS = not significant, Wilcoxon rank sum test). See S1 Data for relative abundance data.
(PDF)

S2 Fig Effects of tetracycline treatment on survivorship, presented as the total number and percent of bees surviving on Day 3 post-treatment. A) Number of bees recovered from hive experiment 2. B) Survivorship in lab exposed recovery experiment. C) Survivorship in lab sterile recovery experiment bees. D) Survivorship in lab germ-free bees. *** = P<0.0001, NS = not significant, Chi-squared test. See S2 Data for survival counts.
(PDF)

S3 Fig Effects of tetracycline treatment on absolute abundances of five members of the core bee gut community, on Day 3, Day 5, and Day 7 post-treatment. Boxplots show that tetracycline exposure lowered abundances of these groups, as estimated by qPCR, at all time points post-treatment. A) *Bifidobacterium*, B) *Lactobacillus* Firm-5, C) *Lactobacillus* Firm-4, D) *Snodgrassella*, E) *Bartonella*. ** = P<0.001, *** = P<0.0001, Wilcoxon rank sum tests. See S1 Data for absolute abundance data.
(PDF)

S4 Fig Effects of tetracycline treatment on absolute abundances of five bacterial groups, on Day 3, Day 5, and Day 7 post-treatment. Boxplots show shifts in abundance, as estimated by qPCR, at at least one time point post-treatment. A-B) Two core bee gut bacteria (Alpha 2.1 and *Frischella*) decreased in abundance following tetracycline treatment, C) The environmental bacterium *Lactobacillus kunkeei* decreased at Days 3 and 5. D-E) Two opportunistic bacteria, an unclassified member of the Halomonadaceae family and *Serratia*, significantly increased following tetracycline treatment, at days three and five, respectively. * = P<0.05, ** = P<0.001, Wilcoxon rank sum tests. See S1 Data for absolute abundance data.
(PDF)

S5 Fig Effects of tetracycline treatment on gut microbiome composition of bees kept in laboratory growth chambers, at Days 3, 5, and 7 post-treatment. A) Stacked column graphs showing the absolute abundances (qPCR adjusted for rRNA gene copy number) of bacterial species for bees kept in the sterile experimental condition at Day 3 (treatment n = 9, control n = 9), Day 5 (treatment n = 9, control n = 8), and Day 7 (treatment n = 10, control n = 10), see S11 Data for sample details and S12 Data for absolute and relative abundances. Boxplot on the right shows the total 16S rRNA gene copies. Treatment bees contained fewer bacterial cells than control bees at all time points. B) Stacked column graphs showing the absolute abundances of bacterial species for bees kept in the exposed experimental condition at Day 3 (treatment n = 9, control n = 10), Day 5 (treatment n = 9, control n = 9), and Day 7 (treatment n = 8, control n = 10), see S11 Data for sample details and S13 Data for absolute and relative abundances. Boxplot on the right shows the total 16S rRNA gene copies. Treatment bees contained fewer bacterial cells than control bees on Days 3 and 5 post-treatment. * = P<0.05, ** = P<0.001, Wilcoxon rank sum tests.
(PDF)

S6 Fig Screen for fungi in hive recovery bees. A) Percent of hive experiment bees positive for fungi based on diagnostic PCR with universal fungal primers at Days 0, 3, 5, and 7 post-treatment. On Day 0 none of the samples were positive for fungi. On Day 3, four treatment bees (26%) were positive for fungi, and on Day 5 eight treatment bees (53%) were positive for fungi, and on Day 7 two treatment bees (13%) were positive for fungi based on diagnostic PCR. One control bee was positive for fungi on Day 7. B) Identity of the randomly sequenced clones from treatment and control bees.
(PDF)

S7 Fig The average relative abundances of bacterial species present in control and treatment bees at Days 3, 5, and 7 post-treatment. See S1 Data for relative abundance data.
(PDF)

S8 Fig Effects of tetracycline treatment on relative abundances for bacterial species in which treatment had a significant effect. * = P<0.05, ** = P<0.001, Wilcoxon rank sum tests. See S1 Data for relative abundance data.
(PDF)

S9 Fig Principal coordinate analysis. Principal coordinate analysis (weighted and unweighted Unifrac) of the gut microbiome composition in control and treatment bees kept in A-B) the sterile experimental condition or C-D) the exposed experimental condition at Days 0, 3, 5, and 7 post-treatment. See S14 Data for alpha and beta diversity data.
(PDF)

S1 Table Number of 16S rRNA operons per genome for each of the bacteria detected in this study. (PDF)

S1 Data Relative and absolute abundance data for hive recovery bees. (XLSX)

S2 Data Day three survival counts for Fig 2A and S2A–S2D Fig. (XLSX)

S3 Data Alpha and beta diversity data for Fig 3. (XLSX)

S4 Data Number of OTUs at 99% identity threshold for each taxon. (XLSX)

S5 Data Stats for Fig 5B. (XLSX)

S6 Data Stats for Fig 5D. (XLSX)

S7 Data Survival counts for *Serratia* infection experiment (age-controlled workers). (XLSX)

S8 Data Survival counts for *Serratia* infection experiment (non age-controlled workers). (XLSX)

S9 Data Stats for Fig 6B. (XLSX)

S10 Data Survival counts for immunity challange experiment. (XLSX)

S11 Data Sample information. (XLSX)

S12 Data Absolute and relative abundance data for lab sterile recovery bees. (XLSX)

S13 Data Absolute and relative abundance data for lab exposed recovery bees. (XLSX)

S14 Data Alpha and beta diversity data for S9 Fig. (XLSX)

## Abbreviations

AFB: American Foulbrood
CCD: colony collapse disorder
OTU: operational taxonomic unit
*tetL*: tetracycline resistance gene
