# Supplementary figures and images for: Antibiotic exposure perturbs the gut microbiota and elevates mortality in honeybees
Source: PLoS Biol. 2017 Mar 14;15(3):e2001861. doi: 10.1371/journal.pbio.2001861 (PMC5349420; doi:10.1371/journal.pbio.2001861)

**A**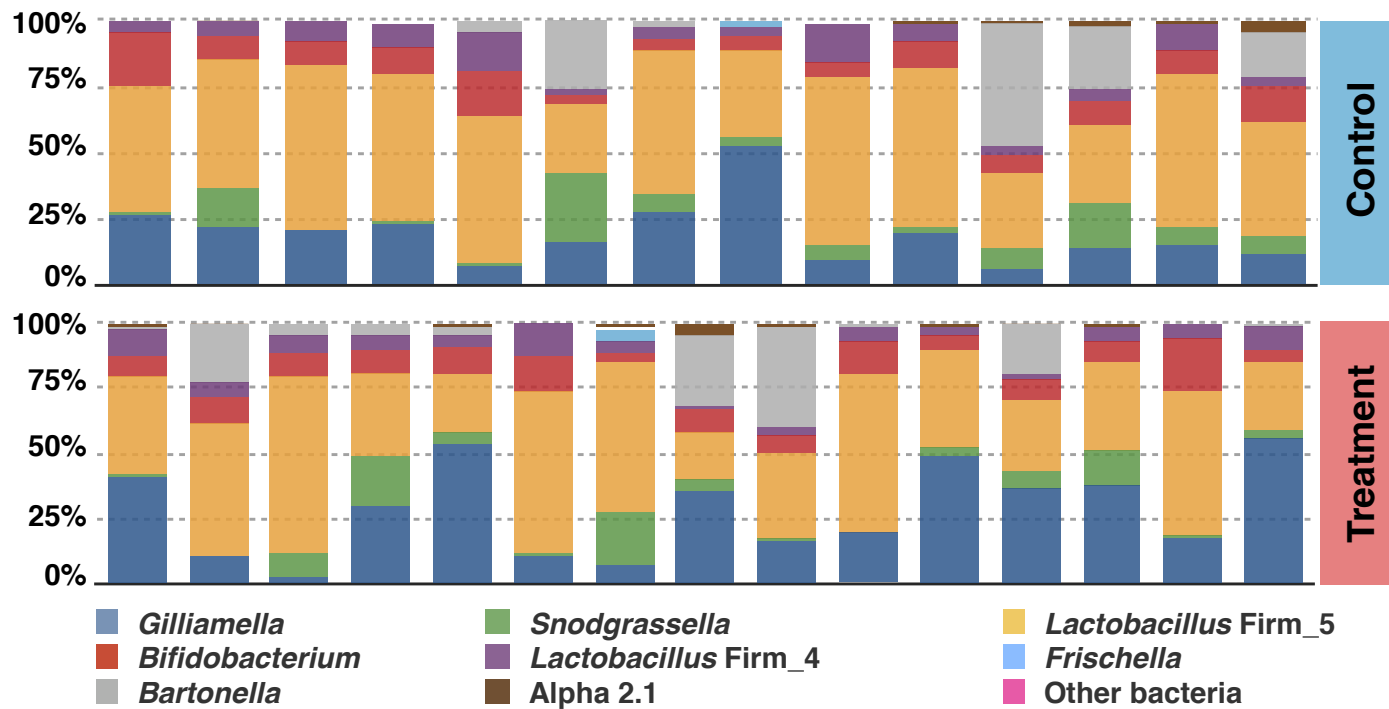**B**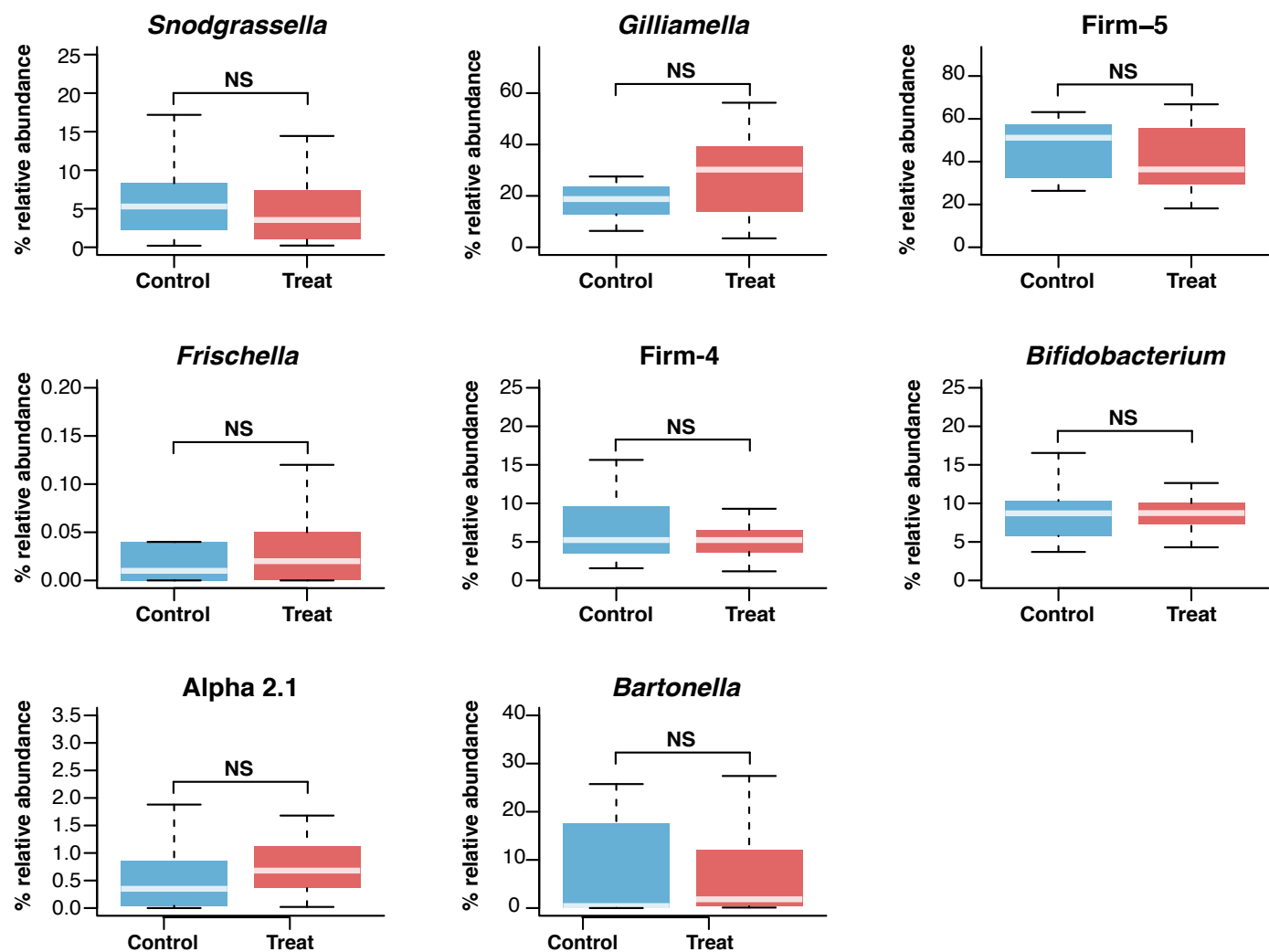

Supplement: S1 Fig — A) Stacked column graphs showing the relative abundances of bee gut bacterial species in control bees (n = 14) and treatment bees (n = 15) after five days of tetracycline treatment (Day 0 post-treatment), see Dataset S7 for sample details. B) Boxplots showing the relative abundances of the eight core bee gut species in control and treatment bees on Day 0. None of the eight core species showed significant changes in relative abundance following tetracycline exposure at Day 0 (NS = not significant, Wilcoxon rank sum test). See S1 Data for relative abundance data. (PDF) [file pbio.2001861.s001.pdf]

**A****Hive Experiment 2**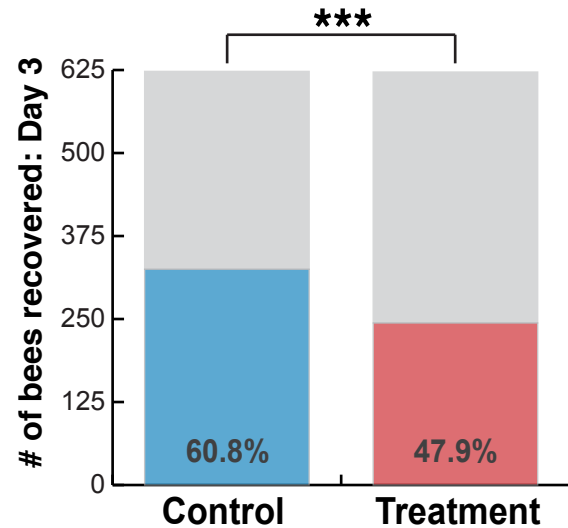**B****Lab Exposed**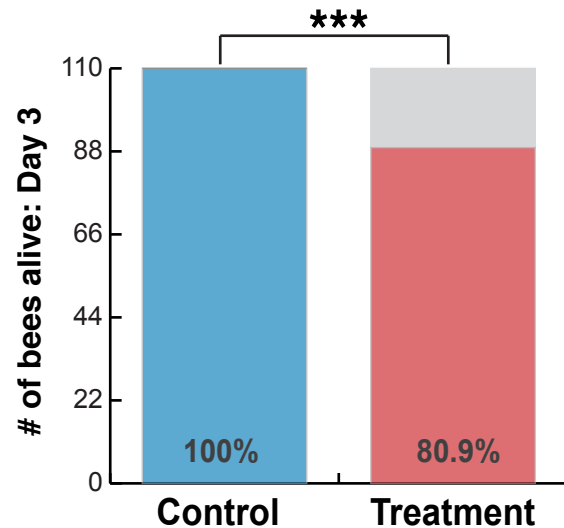**C****Lab Sterile**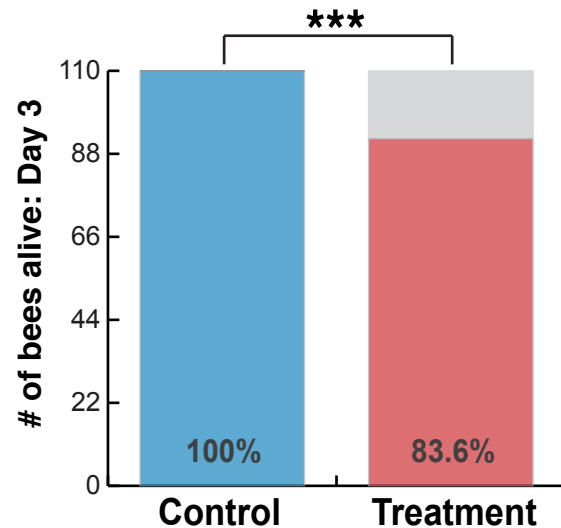**D****Lab Germ-free**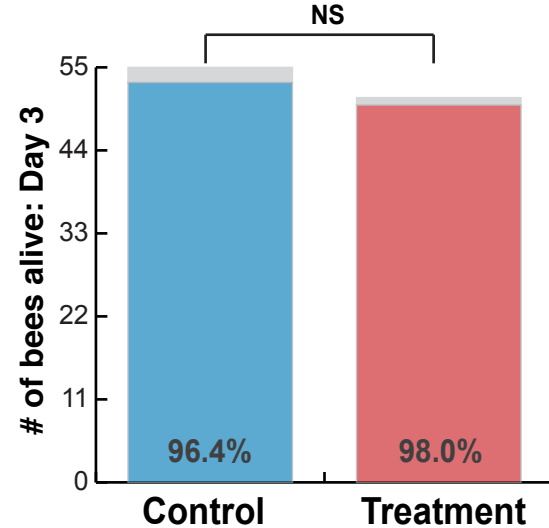

Supplement: S2 Fig — A) Number of bees recovered from hive experiment 2. B) Survivorship in lab exposed recovery experiment. C) Survivorship in lab sterile recovery experiment bees. D) Survivorship in lab germ-free bees. *** = P<0.0001, NS = not significant, Chi-squared test. See S2 Data for survival counts. (PDF) [file pbio.2001861.s002.pdf]

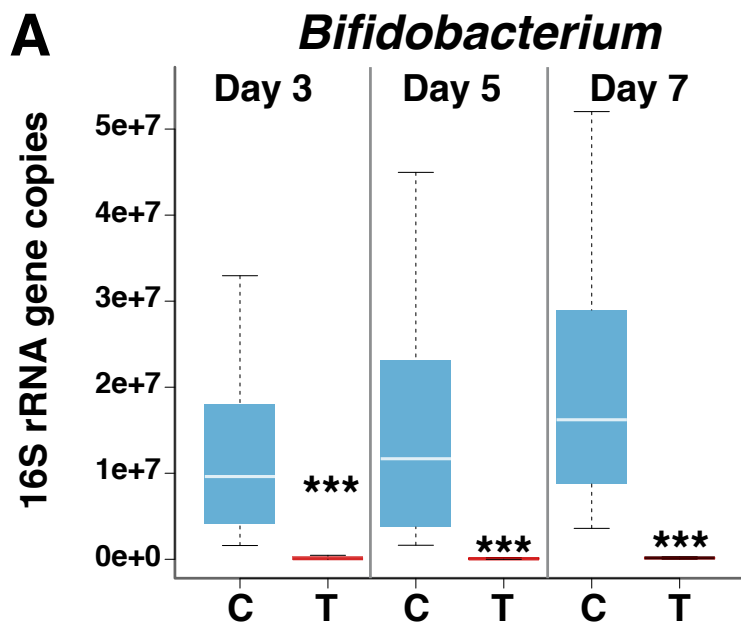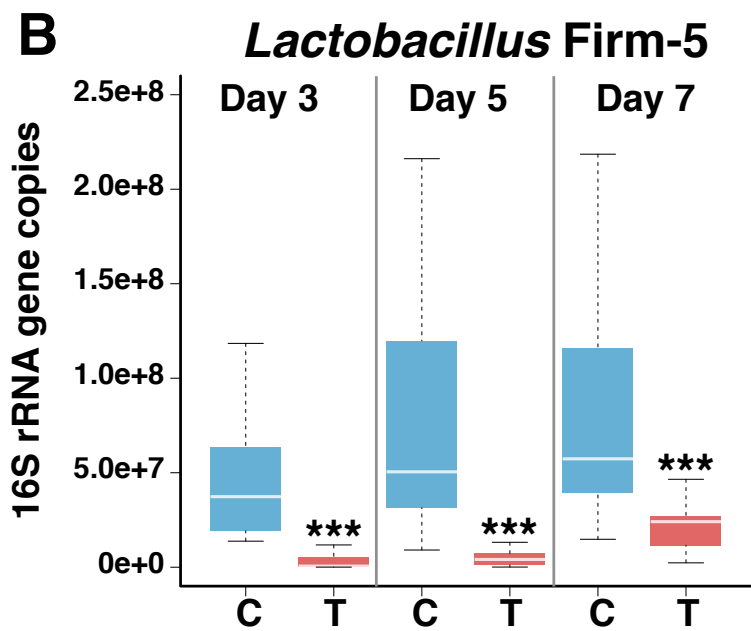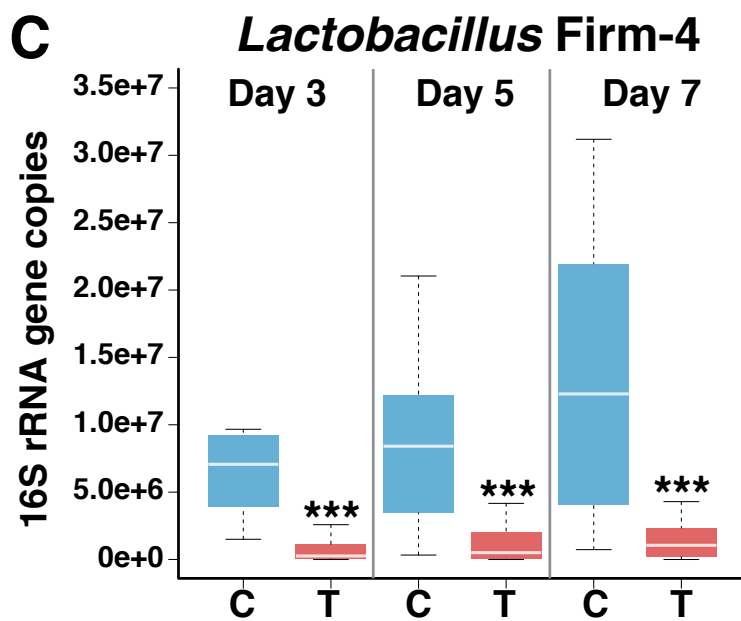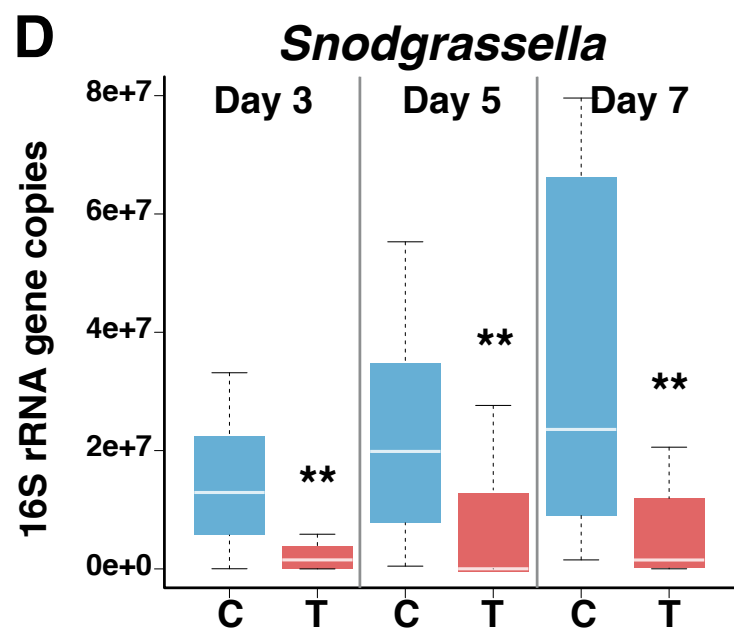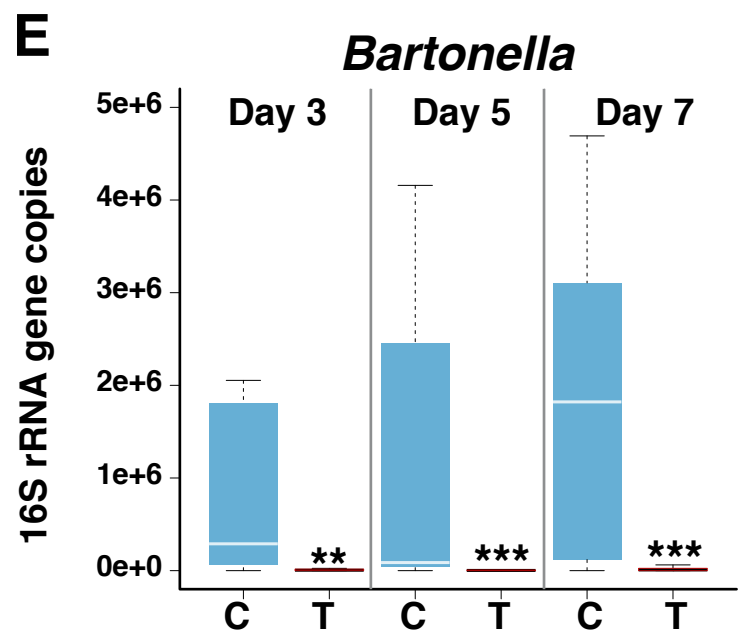

Supplement: S3 Fig — Boxplots show that tetracycline exposure lowered abundances of these groups, as estimated by qPCR, at all time points post-treatment. A) Bifidobacterium, B) Lactobacillus Firm-5, C) Lactobacillus Firm-4, D) Snodgrassella, E) Bartonella. ** = P<0.001, *** = P<0.0001, Wilcoxon rank sum tests. See S1 Data for absolute abundance data. (PDF) [file pbio.2001861.s003.pdf]

**A****Alpha 2.1**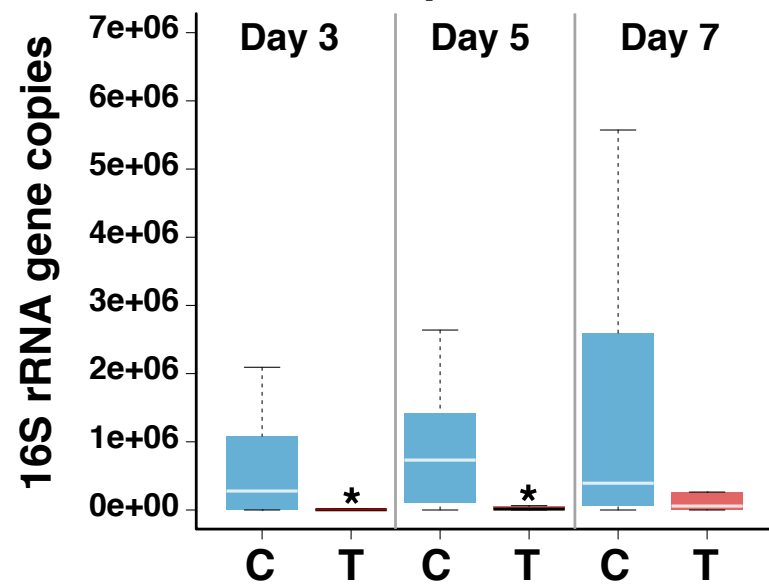**B*****Frischella***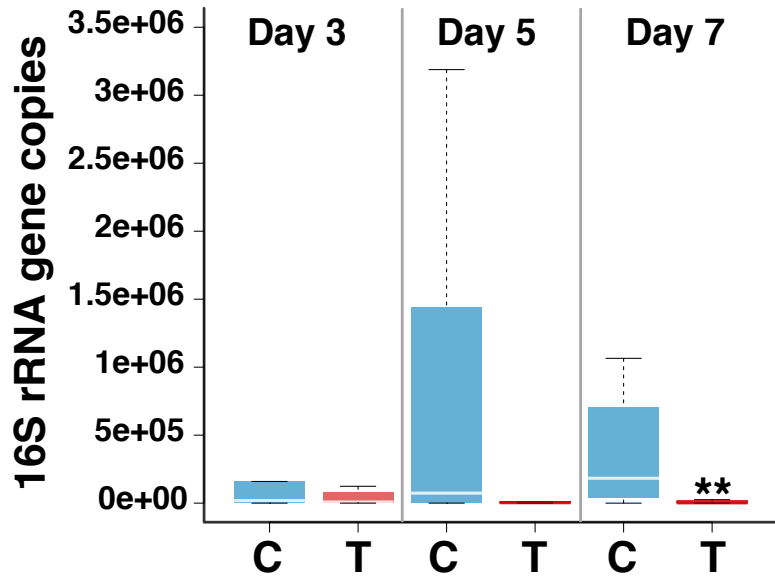**C*****Lactobacillus kunkeei***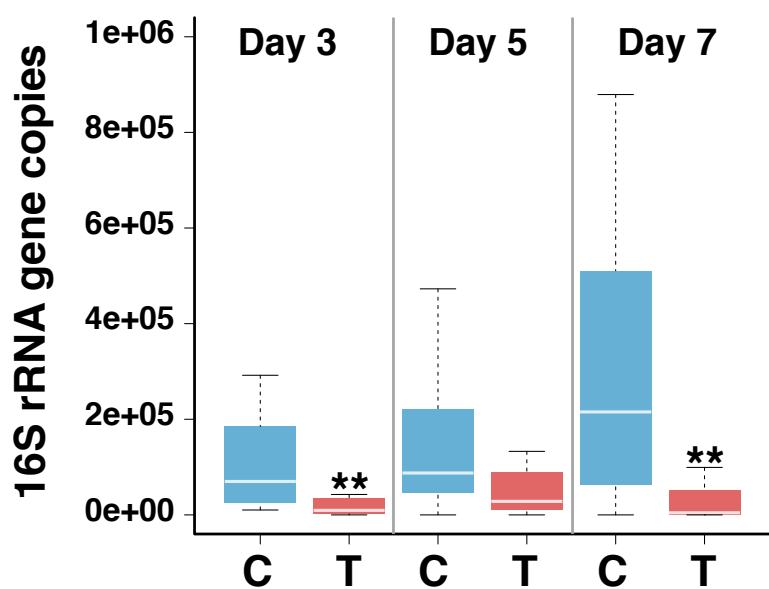**D*****Halomonadaceae***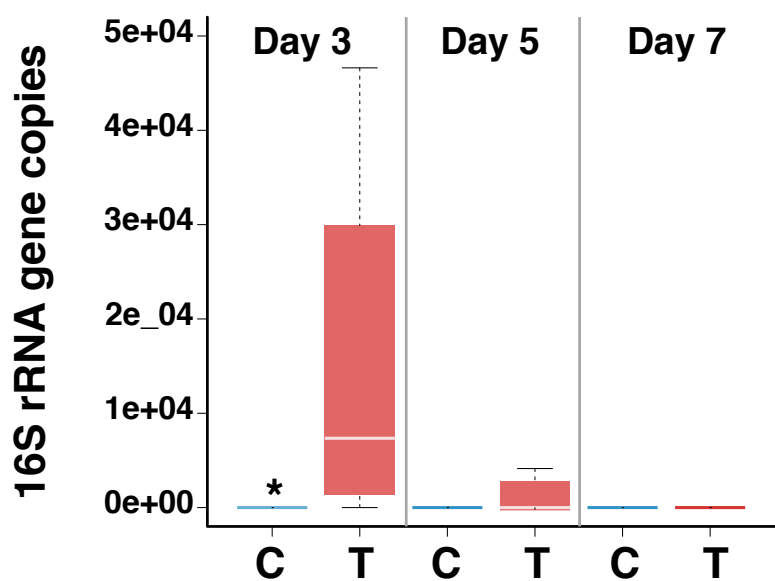**E*****Serratia***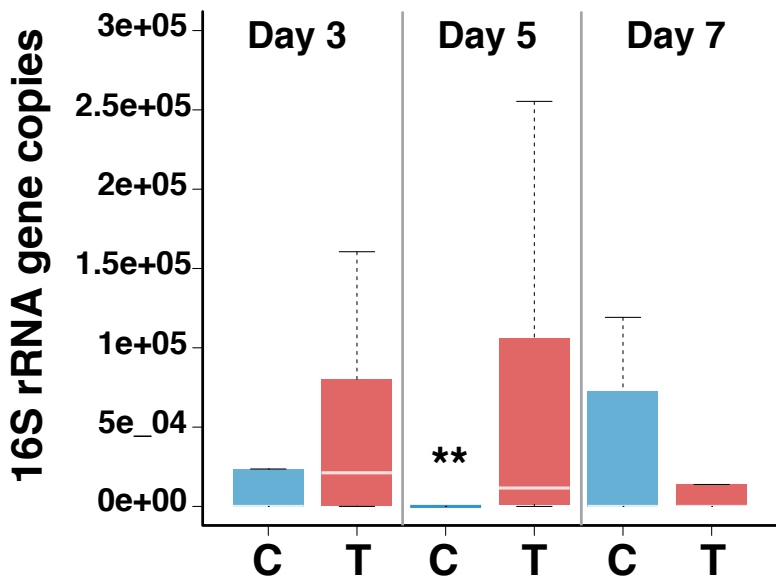

Supplement: S4 Fig — Boxplots show shifts in abundance, as estimated by qPCR, at at least one time point post-treatment. A-B) Two core bee gut bacteria (Alpha 2.1 and Frischella) decreased in abundance following tetracycline treatment, C) The environmental bacterium Lactobacillus kunkeei decreased at Days 3 and 5. D-E) Two opportunistic bacteria, an unclassified member of the Halomonadaceae family and Serratia, significantly increased following tetracycline treatment, at days three and five, respectively. * = P<0.05, ** = P<0.001, Wilcoxon rank sum tests. See S1 Data for absolute abundance data. (PDF) [file pbio.2001861.s004.pdf]

**A****Lab sterile recovery**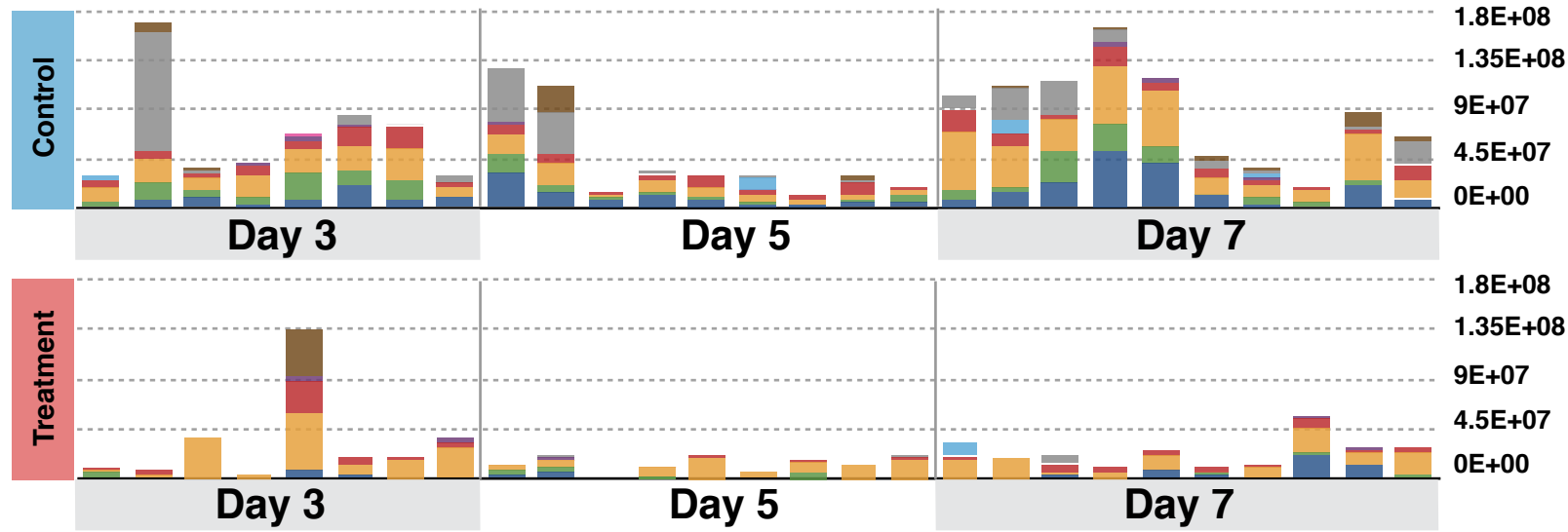**All bacteria**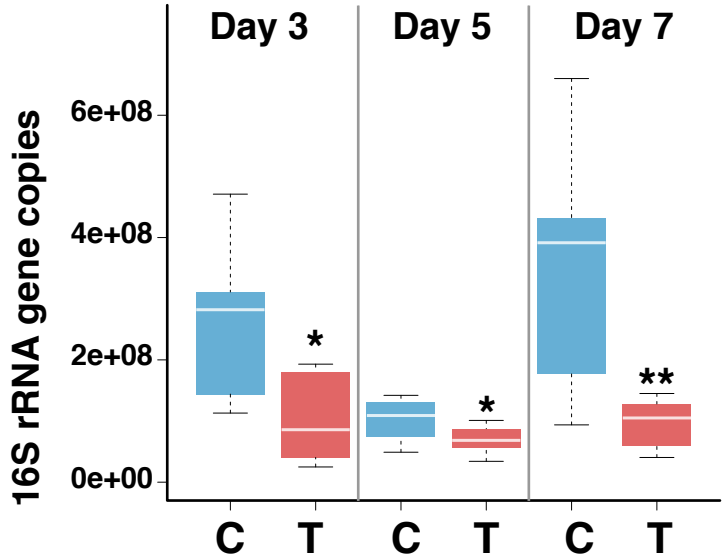**B****Lab exposed recovery**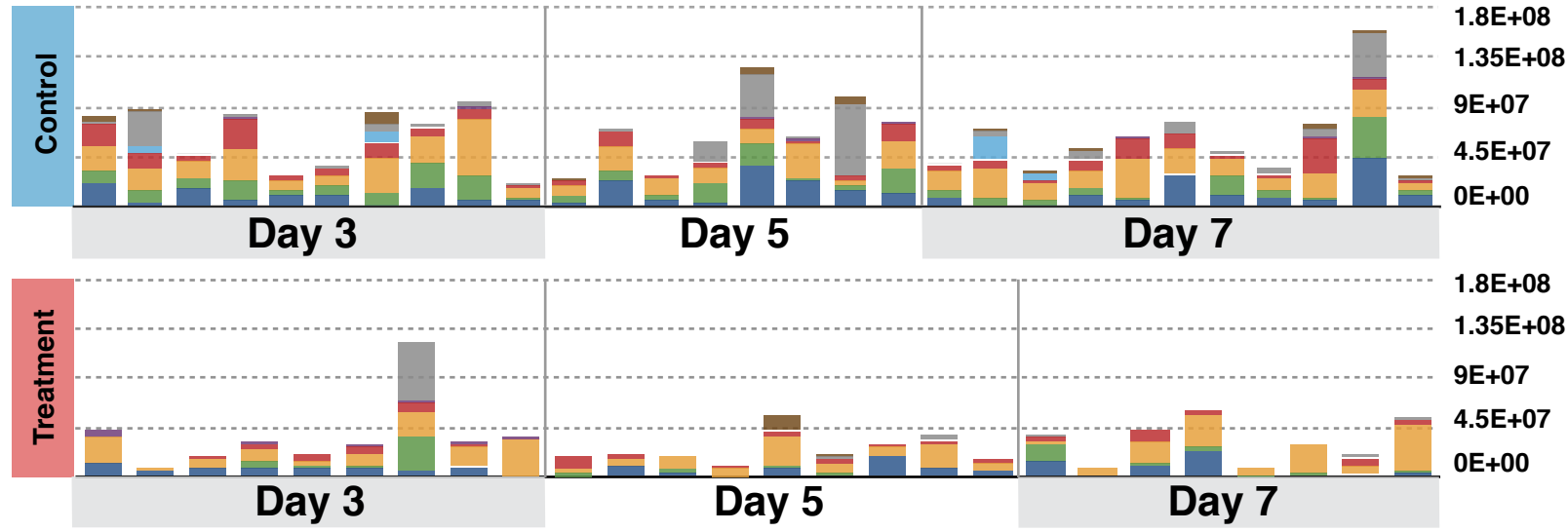**All bacteria**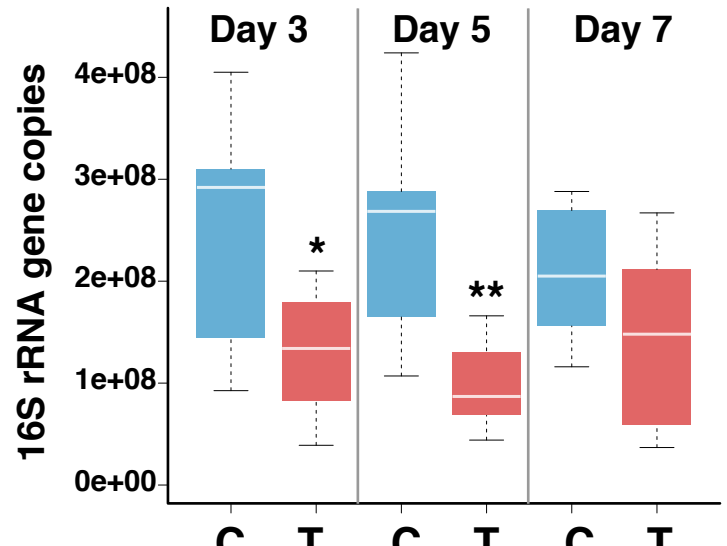

Supplement: S5 Fig — A) Stacked column graphs showing the absolute abundances (qPCR adjusted for rRNA gene copy number) of bacterial species for bees kept in the sterile experimental condition at Day 3 (treatment n = 9, control n = 9), Day 5 (treatment n = 9, control n = 8), and Day 7 (treatment n = 10, control n = 10), see S11 Data for sample details and S12 Data for absolute and relative abundances. Boxplot on the right shows the total 16S rRNA gene copies. Treatment bees contained fewer bacterial cells than control bees at all time points. B) Stacked column graphs showing the absolute abundances of bacterial species for bees kept in the exposed experimental condition at Day 3 (treatment n = 9, control n = 10), Day 5 (treatment n = 9, control n = 9), and Day 7 (treatment n = 8, control n = 10), see S11 Data for sample details and S13 Data for absolute and relative abundances. Boxplot on the right shows the total 16S rRNA gene copies. Treatment bees contained fewer bacterial cells than control bees on Days 3 and 5 post-treatment. * = P<0.05, ** = P<0.001, Wilcoxon rank sum tests. (PDF) [file pbio.2001861.s005.pdf]
