# Supplementary material for: Antibiotic exposure perturbs the gut microbiota and elevates mortality in honeybees
Source: PLoS Biol. 2017 Mar 14;15(3):e2001861. doi: 10.1371/journal.pbio.2001861 (PMC5349420; doi:10.1371/journal.pbio.2001861)
Supplement: S6 Fig — A) Percent of hive experiment bees positive for fungi based on diagnostic PCR with universal fungal primers at Days 0, 3, 5, and 7 post-treatment. On Day 0 none of the samples were positive for fungi. On Day 3, four treatment bees (26%) were positive for fungi, and on Day 5 eight treatment bees (53%) were positive for fungi, and on Day 7 two treatment bees (13%) were positive for fungi based on diagnostic PCR. One control bee was positive for fungi on Day 7. B) Identity of the randomly sequenced clones from treatment and control bees. (PDF) [file pbio.2001861.s006.pdf]

A

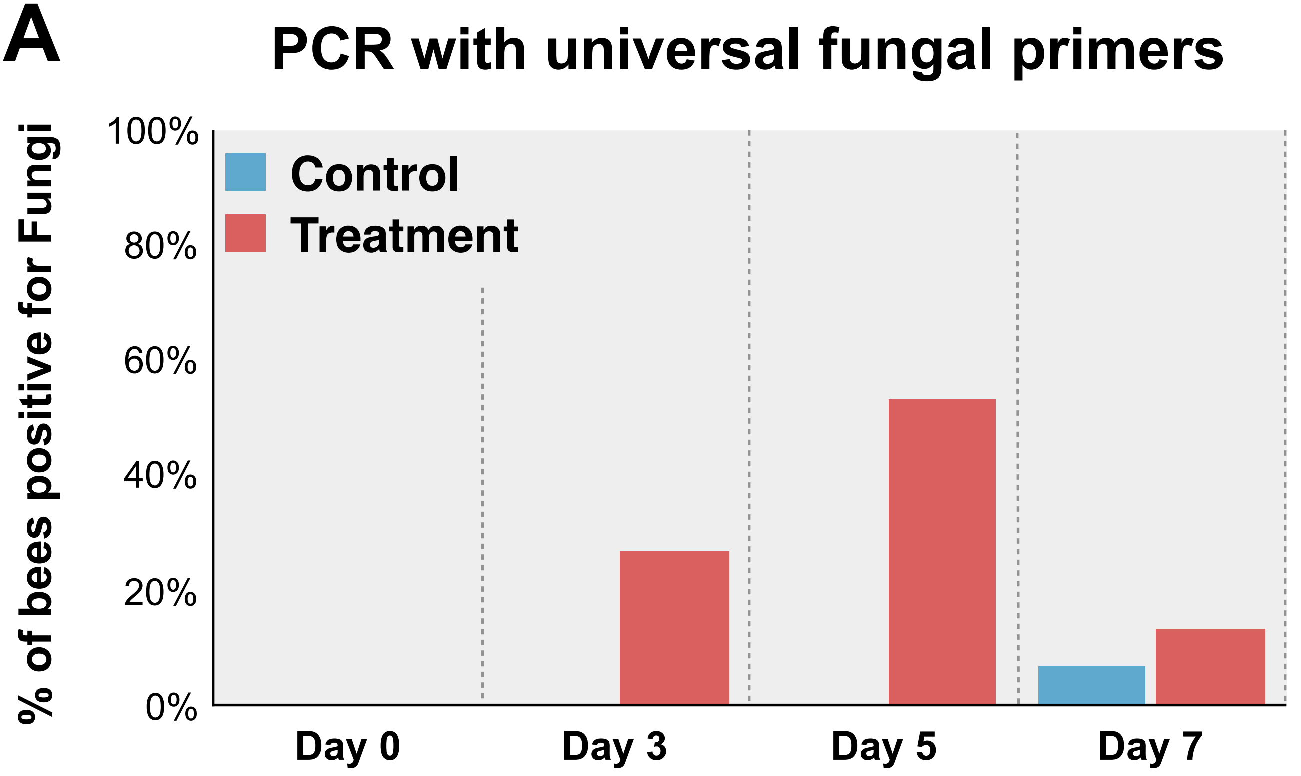

B

Clones	Sample	Species*	Kingdom
T2 D3 1	Treatment bee 2 day 3	*Metschnikowia koreensis*	Fungi
T2 D3 2	Treatment bee 2 day 3	*Metschnikowia koreensis*	Fungi
T2 D3 3	Treatment bee 2 day 3	*Metschnikowia koreensis*	Fungi
T2 D3 1	Treatment bee 2 day 3	*Metschnikowia koreensis*	Fungi
T6 D3 1	Treatment bee 6 day 3	*Lachancea thermotolerans*	Fungi
T3 D5 1	Treatment bee 3 day 5	*Starmerella meliponinorum*	Fungi
T10 D5 1	Treatment bee 10 day 5	*Nothoscordum bivalve*	Plantae
T10-D5 2	Treatment bee 10 day 5	*Nothoscordum texanum*	Plantae
T13 D5 1	Treatment bee 13 day 5	*Metschnikowia koreensis*	Fungi
T1 D7 1	Treatment bee 1 day 7	*Elaeagnus macrophylla*	Plantae
C1 D7 1	Control bee 1 day 7	*Nothoscordum bivalve*	Plantae
C1 D7 2	Control bee 1 day 7	*Nothoscordum bivalve*	Plantae
C1 D7 3	Control bee 1 day 7	*Nothoscordum bivalve*	Plantae
C1 D7 4	Control bee 1 day 7	*Nothoscordum bivalve*	Plantae
C1 D7 5	Control bee 1 day 7	*Nothoscordum bivalve*	Plantae
C1 D7 6	Control bee 1 day 7	*Nothoscordum bivalve*	Plantae
C1 D7 7	Control bee 1 day 7	*Nothoscordum bivalve*	Plantae

* signifies >97% sequence identity to the given species
