# Supplementary material for: Antibiotic exposure perturbs the gut microbiota and elevates mortality in honeybees
Source: PLoS Biol. 2017 Mar 14;15(3):e2001861. doi: 10.1371/journal.pbio.2001861 (PMC5349420; doi:10.1371/journal.pbio.2001861)
Supplement: S7 Fig — See S1 Data for relative abundance data. (PDF) [file pbio.2001861.s007.pdf]

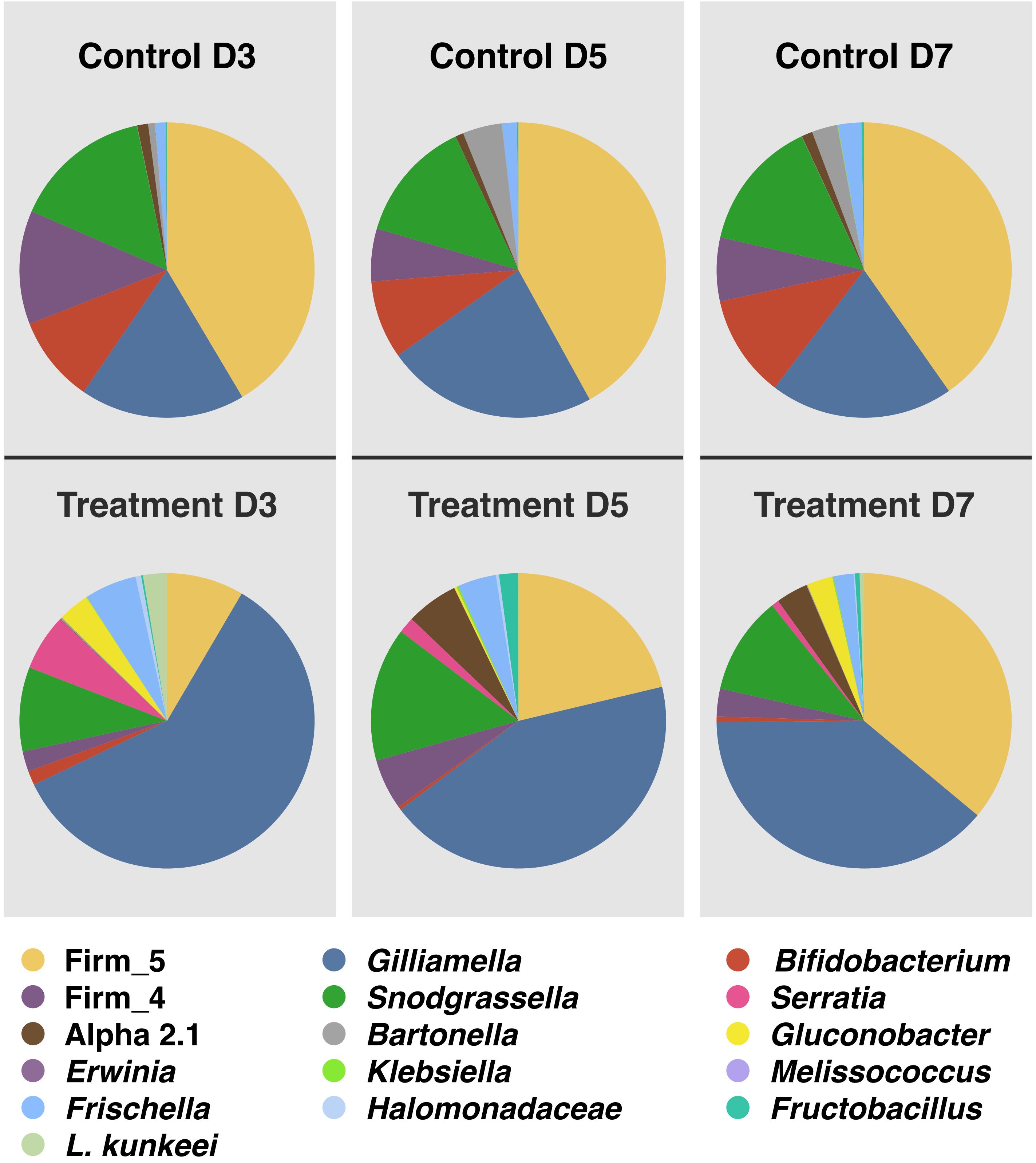
Control D3
Control D5
Control D7
Treatment D3
Treatment D5
Treatment D7
Firm_5
Firm_4
Alpha 2.1
Erwinia
Frischella
L. kunkeei
Gilliamella
Snodgrassella
Bartonella
Klebsiella
Halomonadaceae
Bifidobacterium
Serratia
Gluconobacter
Melissococcus
Fructobacillus
