# Supplementary material for: Antibiotic exposure perturbs the gut microbiota and elevates mortality in honeybees
Source: PLoS Biol. 2017 Mar 14;15(3):e2001861. doi: 10.1371/journal.pbio.2001861 (PMC5349420; doi:10.1371/journal.pbio.2001861)
Supplement: S8 Fig — * = P<0.05, ** = P<0.001, Wilcoxon rank sum tests. See S1 Data for relative abundance data. (PDF) [file pbio.2001861.s008.pdf]

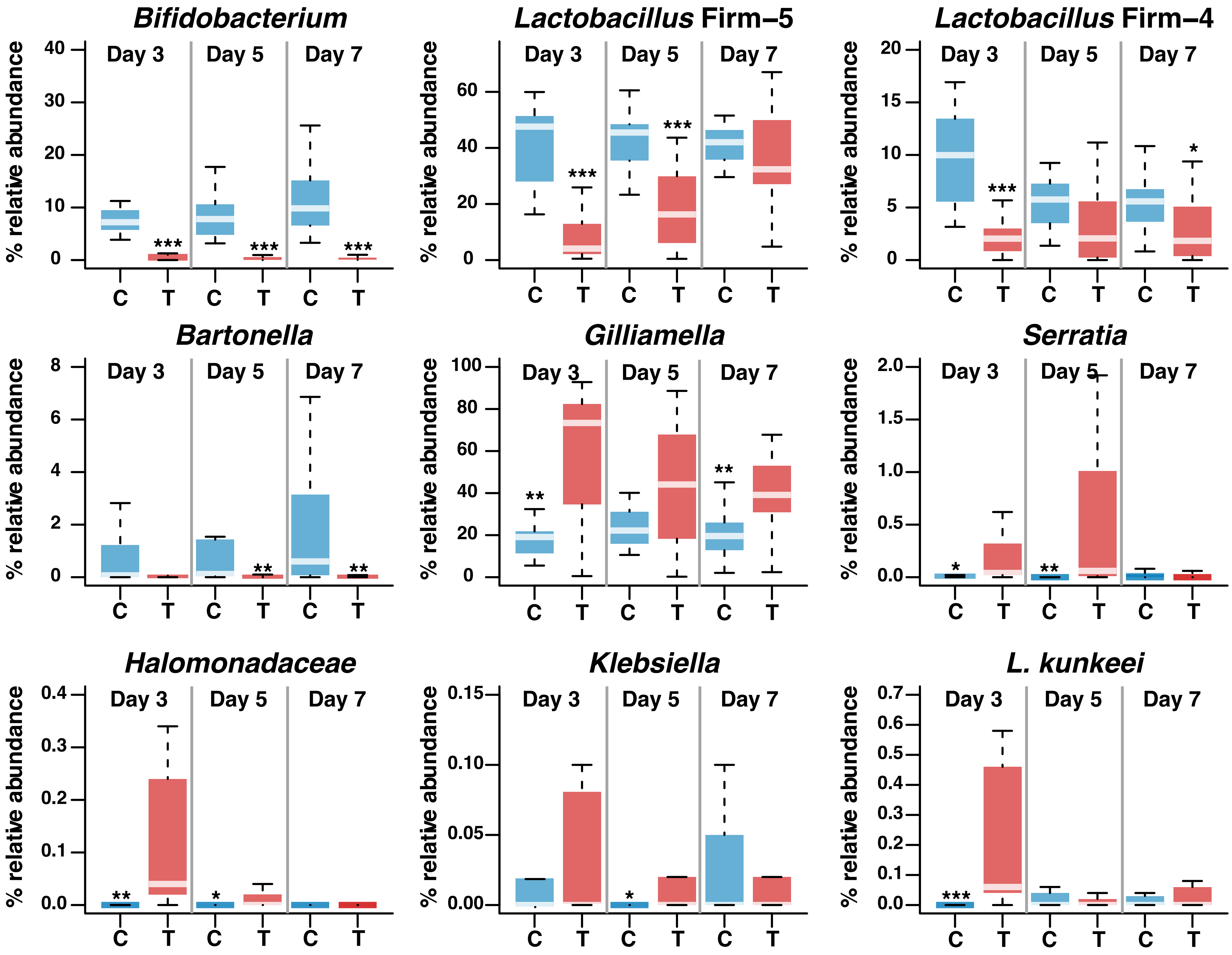

Bifidobacterium
Lactobacillus Firm-5
Lactobacillus Firm-4
Bartonella
Gilliamella
Serratia
Halomonadaceae
Klebsiella
L. kunkeei
% relative abundance
Day 3
Day 5
Day 7
C
T
