# Supplementary material for: Antibiotic exposure perturbs the gut microbiota and elevates mortality in honeybees
Source: PLoS Biol. 2017 Mar 14;15(3):e2001861. doi: 10.1371/journal.pbio.2001861 (PMC5349420; doi:10.1371/journal.pbio.2001861)
Supplement: S9 Fig — Principal coordinate analysis (weighted and unweighted Unifrac) of the gut microbiome composition in control and treatment bees kept in A-B) the sterile experimental condition or C-D) the exposed experimental condition at Days 0, 3, 5, and 7 post-treatment. See S14 Data for alpha and beta diversity data. (PDF) [file pbio.2001861.s009.pdf]

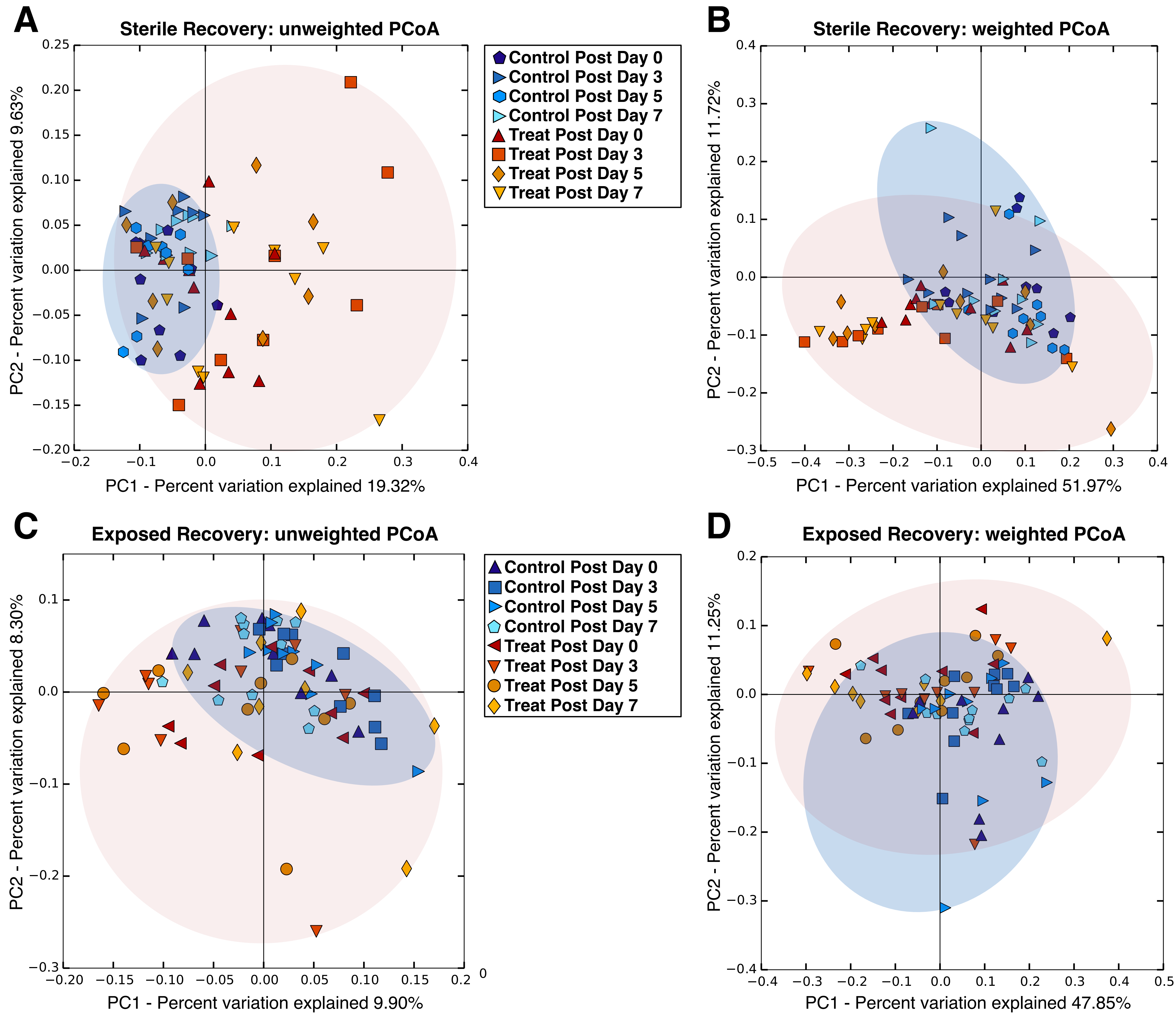
A
Sterile Recovery: unweighted PCoA
PC1 - Percent variation explained 19.32%
PC2 - Percent variation explained 9.63%
Control Post Day 0
Control Post Day 3
Control Post Day 5
Control Post Day 7
Treat Post Day 0
Treat Post Day 3
Treat Post Day 5
Treat Post Day 7
B
Sterile Recovery: weighted PCoA
PC1 - Percent variation explained 51.97%
PC2 - Percent variation explained 11.72%
C
Exposed Recovery: unweighted PCoA
PC1 - Percent variation explained 9.90%
PC2 - Percent variation explained 8.30%
Control Post Day 0
Control Post Day 3
Control Post Day 5
Control Post Day 7
Treat Post Day 0
Treat Post Day 3
Treat Post Day 5
Treat Post Day 7
D
Exposed Recovery: weighted PCoA
PC1 - Percent variation explained 47.85%
PC2 - Percent variation explained 11.25%
