# Supplementary material for: Antibiotic exposure perturbs the gut microbiota and elevates mortality in honeybees
Source: PLoS Biol. 2017 Mar 14;15(3):e2001861. doi: 10.1371/journal.pbio.2001861 (PMC5349420; doi:10.1371/journal.pbio.2001861)
Supplement: S1 Table — (PDF) [file pbio.2001861.s010.pdf]

Table S1. Number of 16S rRNA operons per genome for each of the bacteria detected in this study.

Taxon	Reference	16S rRNA gene copy number*
Gilliamella	*Gilliamella apicola* wkB1	4
Snodgrassella	*Snodgrassella alvi* wkB2	4
Lactobacillus Firm-5	*Lactobacillus sp.* wkB8	4
Bifidobacterium	*Bifidobacterium asteroides* PRL2011	2
Lactobacillus Firm-4	*Lactobacillus* (genus)	**4**
Frischella	*Frischella perrara* PEB0191	4
Bartonella	*Bartonella* (genus)	**2**
Alpha 2.1	*Acetobacteraceae* (family)	**4**
Fructobacillus	*Leuconostocaceae* (family)	**4**
Serratia	*Serratia* (genus)	**7**
Erwinia	*Erwinia* (genus)	**7**
Klebsiella	*Klebsiella* (genus)	**8**
Melissococcus	*Melissococcus* (genus)	**4**
Zymobacter	*Zymobacter* (genus)	**1**
Lactobacillus kunkeei	*Lactobacillus* (genus)	**4**
Gluconobacter	*Gluconobacter* (genus)	**4**

**Operon number was determined using the reference genome for a given bee gut bacterial species when available. For numbers in bold, a complete genome was not available for the species, so the mean 16S rRNA operon copy for the bacterial genus or family was obtained from the rrnDB (https://rrndb.umms.med.umich.edu/) and used as an estimate of operon number.*
